# Do genome-scale models need exact solvers or clearer standards?

**DOI:** 10.15252/msb.20156157

**Published:** 2015-10-14

**Authors:** Ali Ebrahim, Eivind Almaas, Eugen Bauer, Aarash Bordbar, Anthony P Burgard, Roger L Chang, Andreas Dräger, Iman Famili, Adam M Feist, Ronan MT Fleming, Stephen S Fong, Vassily Hatzimanikatis, Markus J Herrgård, Allen Holder, Michael Hucka, Daniel Hyduke, Neema Jamshidi, Sang Yup Lee, Nicolas Le Novère, Joshua A Lerman, Nathan E Lewis, Ding Ma, Radhakrishnan Mahadevan, Costas Maranas, Harish Nagarajan, Ali Navid, Jens Nielsen, Lars K Nielsen, Juan Nogales, Alberto Noronha, Csaba Pal, Bernhard O Palsson, Jason A Papin, Kiran R Patil, Nathan D Price, Jennifer L Reed, Michael Saunders, Ryan S Senger, Nikolaus Sonnenschein, Yuekai Sun, Ines Thiele

**Affiliations:** 1 Department of Bioengineering, University of CaliforniaSan Diego, CA, USA; 2 Department of Biotechnology, Norwegian University of Science and Technology (NTNU)Trondheim, Norway; 3 Luxembourg Centre for Systems Biomedicine, University of LuxembourgBelval, Luxembourg; 4 Sinopia Biosciences Inc.San Diego, CA, USA; 5 Genomatica, Inc.San Diego, CA, USA; 6 Department of Systems Biology, Harvard Medical SchoolBoston, MA, USA; 7 Center for Bioinformatics Tuebingen (ZBIT), University of TuebingenTübingen, Germany; 8 Intrexon, Inc.San Diego, CA, USA; 9 Department of Chemical and Life Science Engineering, Virginia Commonwealth UniversityRichmond, VA, USA; 10 Laboratory of Computational Systems Biotechnology, Ecole Polytechnique Fédérale de LausanneLausanne, Switzerland; 11 The Novo Nordisk Foundation Center for Biosustainability, Technical University of DenmarkLyngby, Denmark; 12 Department of Mathematics, Rose-Hulman Institute of TechnologyTerre Haute, IN, USA; 13 Department of Computing and Mathematical Science, California Institute of TechnologyPasadena, CA, USA; 14 Department of Biological Engineering, Utah State UniversityLogan, UT, USA; 15 Department of Radiology, University of CaliforniaLos Angeles, CA, USA; 16 Institute of Engineering in Medicine, University of CaliforniaSan Diego, CA, USA; 17 Department of Chemical and Biomolecular Engineering (BK21 Plus Program), Korea Advanced Institute of Science and Technology (KAIST)Daejeon, Korea; 18 Babraham InstituteCambridge, UK; 19 Department of Pediatrics, University of CaliforniaSan Diego, CA, USA; 20 Department of Management Science and Engineering, Stanford UniversityStanford, CA, USA; 21 Department of Chemical Engineering and Applied Chemistry, University of TorontoToronto, Ontario, Canada; 22 Department of Chemical Engineering, Pennsylvania State UniversityUniversity Park, PA, USA; 23 Biosciences and Biotechnology Division, Lawrence Livermore National LaboratoryLivermore, CA, USA; 24 Department of Biology and Biological Engineering, Chalmers University of TechnologyGothenburg, Sweden; 25 Australian Institute for Bioengineering & Nanotechnology (AIBN), The University of QueenslandBrisbane, Queensland, Australia; 26 Department of Environmental Biology, Centro de Investigaciones Biológicas (CSIC)Madrid, Spain; 27 Synthetic and Systems Biology Unit, Biological Research CenterSzeged, Hungary; 28 Department of Biomedical Engineering, University of VirginiaCharlottesville, VA, USA; 29 European Molecular Biology LaboratoryHeidelberg, Germany; 30 Institute for Systems BiologySeattle, WA, USA; 31 Department of Chemical and Biological Engineering, University of Wisconsin-MadisonMadison, WI, USA; 32 Department of Biological Systems Engineering, Virginia TechBlacksburg, VA, USA; 33 Institute for Computational and Mathematical Engineering, Stanford UniversityStanford, CA, USA

Constraint-based analysis of genome-scale models (GEMs) arose shortly after the first genome sequences became available. As numerous reviews of the field show, this approach and methodology has proven to be successful in studying a wide range of biological phenomena (McCloskey *et al*, 2013; Bordbar *et al*, 2014). However, efforts to expand the user base are impeded by hurdles in correctly formulating these problems to obtain numerical solutions. In particular, in a study entitled “An exact arithmetic toolbox for a consistent and reproducible structural analysis of metabolic network models” (Chindelevitch *et al*, 2014), the authors apply an exact solver to 88 genome-scale constraint-based models of metabolism. The authors claim that COBRA calculations (Orth *et al*, 2010) are inconsistent with their results and that many published and actively used (Lee *et al*, 2007; McCloskey *et al*, 2013) genome-scale models do support cellular growth in existing studies only because of numerical errors. They base these broad claims on two observations: (i) three reconstructions (*i*AF1260, *i*IT341, and *i*NJ661) compute feasibly in COBRA, but are infeasible when exact numerical algorithms are used by their software (entitled MONGOOSE); (ii) linear programs generated by MONGOOSE for *i*IT341 were submitted to the NEOS Server (a Web site that runs linear programs through various solvers) and gave inconsistent results. They further claim that a large percentage of these COBRA models are actually unable to produce biomass flux. Here, we demonstrate that the claims made by Chindelevitch *et al* (2014) stem from an incorrect parsing of models from files rather than actual problems with numerical error or COBRA computations.

## Calculating numerically accurate and thermodynamically consistent flux states

To prove the feasibility of biomass production in the chosen three models, along with some others, we used the same rational solver QSopt_ex (Applegate *et al*, 2007) to compute feasible flux states. Moreover, we used SymPy, a symbolic math library (Joyner *et al*, 2012), to show that the exactly computed feasible flux state has no numerical error. Furthermore, the computed optimal growth rate from QSopt_ex matched those computed by several floating-point solvers accessed via cobrapy (CPLEX, gurobi, glpk, and MOSEK) and the COBRA toolbox (gurobi and CPLEX) to well within a precision of 10^−6^. Using linear programming problems generated by COBRA for *i*IT341 and a version of the model we constrained to produce no biomass, we observed consistent results between COBRA and the reputable solvers hosted on the NEOS server. These results unequivocally demonstrate that these COBRA models solve consistently with both rational and floating-point solvers. We were able to extend this analysis to show 23 out of 29 models that Chindelevitch *et al* (2014) claim to be “blocked” by FBA have solutions that produce biomass flux without numerical error (Table EV1). Thus, the authors' claim that exact arithmetic is necessary for consistency and reproducibility is inaccurate, along with their findings that these previously published and computed models do not produce biomass flux.

The authors further claim that even more models are “energy blocked” and cannot produce a feasible flux state to produce biomass without thermodynamically infeasible cycles (often referred to as type III loops). Using loopless FBA (Schellenberger *et al*, 2011a), we were able to compute solutions that produce biomass without using these loops. Moreover, we demonstrate that in the case that all reactions allow 0 flux (as is the case in the MONGOOSE formulation), all solutions with loops can be converted into solutions without loops and still produce biomass. As these solutions were obtained using an existing algorithm, the inability of MONGOOSE to identify such solutions is a limitation on the method used by MONGOOSE, not on the published reconstructions as stated by Chindelevitch *et al* (2014). In total, our analysis shows that for 51 out of 59 models, the claims made by MONGOOSE about model blockage are incorrect (Table EV1).

## A call for clear standards in model formulation

While the article by Chindelevitch *et al* (2014) has a valid goal of computing flux states that have been diligently checked for numerical error and thermodynamically infeasible loops, its general conclusions about the current state of COBRA models are incorrect. While more new tools to ensure model quality are welcome, conventional checks with minimal computational overhead already exist, and are routinely employed by the community of flux balance analysis users to ensure that models produce numerically accurate and thermodynamically consistent flux states. We have identified the primary source of the differences between our computations and those reported by Chindelevitch *et al* (2014) to be difficulties with parsing reconstructions from published files and their conversion into computable models. Many of the models were read from reconstructions encoded as SBML files. The mechanism of encoding COBRA model information along with a reconstruction in SBML was originally defined by the COBRA toolbox (Schellenberger *et al*, 2011b), which we therefore consider the reference implementation. For example, as a part of the SBML encoding, boundary metabolites are written with their SBML boundary condition set to true for “exchange” reactions. This convention is meant to signify a system boundary where extracellular metabolites enter and leave the system. The parser developed by Chindelevitch *et al* (2014) to read models from SBML reconstructions ignores this distinction and therefore adds additional constraints to the model. These incorrectly added constraints block any metabolites from entering the system, causing the models to give infeasible growth solutions consistent with mass balance, because mass is not entering and therefore no growth is possible. Thus, erroneous results and conclusions reported by Chindelevitch *et al* (2014) resulted from incorrect parsing of SBML files, resulting in ill-formulated models and a misinterpretation of their calculations.

Part of the issue, however, rests with difficulties associated with encoding models in a consistent format between different labs and software packages. As is the practice in the field, we contacted the authors of the models that we could not solve in order to resolve the differences; after all, the models had been used to perform COBRA computations in their respective publications. In these cases, the authors were able to supply a “fixed” SBML file after correcting errors in the SBML encoding in their respective codebases. An example of one such error was the presence of both “CO2” and “co2” as metabolites in the SBML file for *i*VS941 (Satish Kumar *et al*, 2011). While the GAMS software used in simulating that model is case-insensitive and correctly creates one constraint, parsing the file in other packages (such as the COBRA toolbox, cobrapy, and MONGOOSE) incorrectly created two separate constraints for the uppercase and lowercase versions. Therefore, an inadvertent error in a file-encoding led to different mathematical models in different software tools, and working with the authors of the original model was necessary to resolve the differences. Out of the 88 models attempted by Chindelevitch *et al* (2014), we were able to solve 80, and 9 of these required modifications to fix encoding errors. We attempted to parse 6 of the remaining 8 reconstructions. While the models we parsed from these reconstructions did not solve, this result was still consistent between floating-point and exact solvers.

This situation is a symptom of the well-known issue with interoperability of reconstructions between different laboratories and software packages in constraint-based modeling (Ravikrishnan & Raman, 2015). We believe we can improve upon these issues by better adhering to the standard practices of openness and reproducibility (Dräger & Palsson, 2014). We believe the community needs to standardize on the most recent version of the flux balance constraints (fbc) extension to SBML as the single well-specified format to reliably encode reconstructions, as strict use of fbc version 2 was specifically designed to build genome-scale models unambiguously [SBML-flux Working Group, 2014 SBML Flux Balance Constraints (fbc), http://sbml.org/Documents/Specifications/SBML_Level_3/Packages/Flux_Balance_Constraints_(flux) (Accessed June 13, 2015)]. Therefore, we propose that new reconstructions be published as validated SBML+fbc files and that the authors of existing reconstructions convert them into this format. Moreover, in the interests of reproducibility, studies including flux balance analysis on these genome-scale models should strive to make their code easily reproducible. The models and code used in this study are available as Dataset EV1 and also at https://github.com/opencobra/m_model_collection.

## Supporting Information

Table EV1

Dataset EV1
