# Supplementary material for: Do genome-scale models need exact solvers or clearer standards?
Source: Mol Syst Biol. 2015 Oct 14;11(10):831. doi: 10.15252/msb.20156157 (PMC4631202; doi:10.15252/msb.20156157)
Supplement: Supplementary file 2 — Dataset EV1 [file msb0011-0831-sd2.zip › m_model_collection-master/failed/iPF633.doc]

Synechocystis PCC6803

No.	ORF	gene	enzyme	E.C.	reaction

Glycolysis/Gluconeogenesis

1	sll0593	glk	glucokinase	2.7.1.2	GLC + ATP -> G6P + ADP

2	sll0593	glk	glucokinase	2.7.1.2	bDGLC + ATP <-> bDG6P + ADP

3	sll1709	gdh	glucose dehydrogenase	1.1.1.47	bDGLC + NAD <-> G15LAC + NADH

4	sll1709	gdh	glucose dehydrogenase	1.1.1.47	bDGLC + NADP <-> G15LAC + NADPH

5	slr1608	gdhB	putative glucose dehydrogenase-B periplasmic protein

1.1.5.2	GLC + UBIQ <-> G15LAC + UBIQOL

6	slr1349	pgi	glucose-6-phosphate isomerase	5.3.1.9	G6P <-> F6P

7	slr1349	pgi	glucose-6-phosphate isomerase	5.3.1.9	G6P <-> bDG6P

8	slr1349	pgi	glucose-6-phosphate isomerase	5.3.1.9	bDG6P <-> F6P

9	sll0745	pfkA	phosphofructokinase	2.7.1.11	F6P + ATP -> FDP + ADP

10	sll1196	pfkA	phosphofructokinase	2.7.1.11	UTP + F6P -> UDP + FDP

11	sll1196	pfkA	phosphofructokinase	2.7.1.11	CTP + F6P -> CDP + FDP

12	sll1196	pfkA	phosphofructokinase	2.7.1.11	ITP + F6P -> IDP + FDP

13	sll1196	pfkA	phosphofructokinase	2.7.1.11	ATP + TAG6P -> ADP + TAG16P

14	sll1196	pfkA	phosphofructokinase	2.7.1.11	UTP + TAG6P -> UDP + TAG16P

15	sll1196	pfkA	phosphofructokinase	2.7.1.11	CTP + TAG6P -> CDP + TAG16P

16	sll1196	pfkA	phosphofructokinase	2.7.1.11	ITP + TAG6P -> IDP + TAG16P

17	sll1196	pfkA	phosphofructokinase	2.7.1.11	ATP + S7P -> ADP + S17P

18	sll1196	pfkA	phosphofructokinase	2.7.1.11	UTP + S7P -> UDP + S17P

19	sll1196	pfkA	phosphofructokinase	2.7.1.11	CTP + S7P -> CDP + S17P

20	sll1196	pfkA	phosphofructokinase	2.7.1.11	ITP + S7P -> IDP + S17P

21	slr0952	fbpII, fbp, fdp	fructose-1, 6-bisphosphatase

3.1.3.11	FDP -> F6P + PI

22	slr2094	fbpl, glpX	fructose-1,6-/sedoheptulose-1,7-bisphosphatase

3.1.3.11	3.1.3.37	FDP -> F6P + PI

23	slr2094	fbpl, glpX	fructose-1,6-/sedoheptulose-1,7-bisphosphatase

3.1.3.11	3.1.3.37	S17P -> S7P + PI

24	sll0018	cbbA, cfxA, fbaA, fda	fructose-bisphosphate aldolase class II

4.1.2.13	FDP <-> T3P2 + T3P1

25	slr0943	fda	fructose-bisphosphate aldolase class I

4.1.2.13	S17P <-> T3P2 + E4P

26	slr0783	tpiA	triosephosphate isomerase	5.3.1.1	T3P2 <-> T3P1

27	slr0884	gap1	glyceraldehyde 3-phosphate dehydrogenase 1(NAD+)

1.2.1.12	T3P1 + PI + NAD <-> NADH + 13PDG

28	slr0394	pgk	phosphoglycerate kinase	2.7.2.3	13PDG + ADP <-> 3PG + ATP

29	slr1124	gpmB	phosphoglycerate mutase	5.4.2.1	3PG <-> 2PG

30	slr1945	yibO, pgm	phosphoglycerate mutase	5.4.2.1	3PG <-> 2PG

31	slr1945	yibO, pgm	phosphoglycerate mutase	5.4.2.1	13PDG <-> 23PDG

32	slr0752	eno	enolase	4.2.1.11	2PG <-> PEP

33	sll0587	pyk1	pyruvate kinase1	2.7.1.40	PEP + ADP -> PYR + ATP

34	sll1275	pyk2	pyruvate kinase2	2.7.1.40	PEP + ADP -> PYR + ATP

35	sll1275	pyk2	pyruvate kinase 2	2.7.1.40	UDP + PEP -> UTP + PYR

36	sll1275	pyk2	pyruvate kinase2	2.7.1.40	GDP + PEP -> GTP + PYR

37	sll1275	pyk2	pyruvate kinase 2	2.7.1.40	CDP + PEP -> CTP + PYR

38	sll1275	pyk2	pyruvate kinase 2	2.7.1.40	IDP + PEP -> ITP + PYR

39	sll1275	pyk2	pyruvate kinase 2	2.7.1.40	PEP + DADP -> PYR + DATP

40	sll1275	pyk2	pyruvate kinase 2	2.7.1.40	PEP + DGDP -> PYR + DGTP

41	slr1934	lipo	pyruvate dehydrogenase E1 component alpha subunit

1.2.4.1	PYR + LIPO <-> SADDL + CO2

42	sll1721	pdhB	pyruvate dehydrogenase E1 component beta subunit

1.2.4.1	PYR + COA + NAD -> NADH + CO2 + ACCOA

43	sll1721	pdhB	pyruvate dehydrogenase E1 component beta subunit

1.2.4.1	PYR + TPP -> 2AHTPP + CO2

44	sll1721	pdhB	pyruvate dehydrogenase E1 component beta subunit

1.2.4.1	2AHTPP + LIPO -> TPP + SADDL

45	sll1721	pdhB	pyruvate dehydrogenase E1 component beta subunit

1.2.4.1	PYR + COA + NADP -> NADPH + CO2 + ACCOA

46	sll1841	odhB, citM	dihydrolipoamide acetyltransferase component (E2) of pyruvate dehydrogenase complex

2.3.1.12	SADDL + COA <-> DLIPO + ACCOA

47	slr1096	phdD, lpd	dihydrolipoamide dehydrogenase

1.8.1.4	DLIPO + NAD <-> LIPO + NADH

48	sll0990	fer1	formaldehyde dehydrogenase (glutathione) / alcohol dehydogenase

1.2.1.1	1.1.1.1	ETH + NAD <-> ACAL + NADH

49	slr0942	fer2	aldehyde reductase	1.1.1.2	ETH + NADP <-> ACAL + NADPH

50	slr0091	fer3	aldehyde dehydrogenase	1.2.1.3	AC + NADH <-> ACAL + NAD

51	sll0542	acs	acetyl-coenzyme A synthetase	6.2.1.1	ATP + AC + COA -> AMP + PPI + ACCOA

52	sll0726	pgm	phosphoglucomutase	5.4.2.2	G1P <-> G6P

Citrate cycle (TCA cycle)

53	sll1758	pgm2	phosphoglucomutase	5.4.2.2	G1P <-> G6P

54	sll0401	gltA, gluT	citrate synthase	2.3.3.1	ACCOA + OA -> COA + CIT

55	slr0665	acnB	aconitate hydratase	4.2.1.3	CIT <-> ICIT

56	slr1289	icd	isocitrate dehydrogenase (NADP)	1.1.1.42	ICIT + NADP -> CO2 + NADPH + AKG

57	sll1023	sucC	succinyl-CoA synthetase beta chain	6.2.1.5	SUCCOA + ADP + PI <-> ATP + COA + SUCC

58	sll1557	sucD	succinyl-CoA synthetase alpha chain

6.2.1.5	SUCCOA + ADP + PI <-> ATP + COA + SUCC

59	sll0823	sdhB	succinate dehydrogenase iron-sulfur protein

1.3.99.1	FADH2 + FUM <-> SUCC + FAD

60	sll1625	sdhB	succinate dehydrogenase iron-sulfur protein

1.3.99.1	FADH2 + FUM <-> SUCC + FAD

61	slr1233	frdA	succinate dehydrogenase flavoprotein subunit	1.3.99.1	FADH2 + FUM <-> SUCC + FAD

62	slr0018	fumC	fumarate hydratase	4.2.1.2	FUM <-> MAL

63	sll0891	citH, ldh	malate dehydrogenase	1.1.1.37	MAL + NAD <-> NADH + OA

Pentose phosphate pathway

64	sll1758	r1p	phosphoglucomutase	5.4.2.2	R1P <-> R5P

65	slr1843	zwf	glucose-6-phosphate 1-dehydrogenase

1.1.1.49	G6P + NADP <-> D6PGL + NADPH

66	sll1479	sol	6-phosphogluconolactonase	3.1.1.31	D6PGL -> D6PGC

67	sll0329	gnd	6-phosphogluconate dehydrogenase, decarboxylating

1.1.1.44	D6PGC + NADP <-> NADPH + CO2 + RL5P

68	sll0807	rpe	pentose-5-phosphate-3-epimerase	5.1.3.1	RL5P <-> X5P

69	sll1070	tktA	transketolase	2.2.1.1	R5P + X5P <-> T3P1 + S7P

70	sll1070	tktA	transketolase	2.2.1.1	X5P + E4P <-> F6P + T3P1

71	slr1793	talB, tal	transaldolase	2.2.1.2	T3P1 + S7P <-> E4P + F6P

72	slr0194	rpiA	ribose 5-phosphate isomerase A	5.3.1.6	RL5P <-> R5P

73	ssl2153	rpiB	ribose 5-phosphate isomerase B	5.3.1.6	RL5P <-> R5P

74	sll1776	deoC, dra	deoxyribose-phosphate aldolase

4.1.2.4	DR5P -> ACAL + T3P1

75	sll0469	prsA, prs	ribose-phosphate pyrophosphokinase

2.7.6.1	R5P + ATP <-> PRPP + AMP

76	sll0469	prsA, prs	ribose-phosphate pyrophosphokinase

2.7.6.1	R5P + DATP <-> PRPP + DAMP

Fructose and mannose metabolism

77	slr1448	cscK	fructokinase	2.7.1.4	FRU + ATP -> ADP + F6P

78	sll1496	m1p	mannose-1-phosphate guanylyltransferase / phosphomannomutase

2.7.7.13	5.4.2.8	GTP + MAN1P -> PPI + GDPMAN

79	sll1496	m1p2	mannose-1-phosphate guanylyltransferase / phosphomannomutase

2.7.7.13	5.4.2.8	MAN6P <-> MAN1P

80	slr1334	man6	phosphomannomutase	5.4.2.8	MAN6P <-> MAN1P

81	sll1370	rfbM	GDP-mannose pyrophosphorylase	2.7.7.22	GDPMAN + PI -> MAN1P + GDP

82	slr0493	rfbN	putative mannose-1-phosphate guanylyltransferase

2.7.7.22	GDPMAN + PI -> MAN1P + GDP

83	slr2074	manA, pmi	putative mannose-1-phosphate guanylyltransferase

2.7.7.22	GDPMAN + PI -> MAN1P + GDP

84	sll1558	man2	mannose-1-phosphate guanyltransferase

2.7.7.13	GTP + MAN1P -> PPI + GDPMAN

85	sll1212	rfbD	GDP-D-mannose dehydratase	4.2.1.47	GDPMAN -> GHMAN

86	slr1072	yefA, rfbD	GDP-D-mannose dehydratase

4.2.1.47	GDPMAN -> GHMAN

87	sll1213	rfbd	GDP-L-fucose synthase	1.1.1.271	GHMAN + NADPH -> GDPFUC + NADP

88	sll1213	ghm	GDP-L-fucose synthase	1.1.1.271	GHMAN + NADH -> GDPFUC + NAD

89	sll0018	cbbA	fructose-bisphosphate aldolase class II

4.1.2.13	F1P <-> T3P2 + GLYALDHY

Pyruvate metabolism

90	slr0943	fda	fructose-bisphosphate aldolase class I

4.1.2.13	UDPG + F6PC <-> UDP + S6P

91	sll0244	galE	UDP-glucose 4-epimerase	5.1.3.2	UDPGAL <-> UDPG

92	slr1067	galD	UDP-glucose 4-epimerase	5.1.3.2	UDPGAL <-> UDPG

93	slr1078	galE	UDP-glucose 4-epimerase	5.1.3.2	UDPGAL <-> UDPG

94	sll0542	acs	acetyl-coenzyme A synthetase	6.2.1.1	ACTADEN + COA <-> AMP + ACCOA

95	sll0542	acs	acetyl-coenzyme A synthetase	6.2.1.1	ATP + AC <-> PPI + ACTADEN

96	slr1096	phdD, lpd	dihydrolipoamide dehydrogenase

1.8.1.4	PYR + COA + NAD -> NADH + CO2 + ACCOA

97	sll1299	ackA	acetate kinase	2.7.2.1	ACTP + ADP <-> ATP + AC

98	slr2132	pta	phosphotransacetylase	2.3.1.8	ACCOA + PI <-> ACTP + COA

99	slr0435	accB, fabE	acetyl-CoA carboxylase biotin carboxyl carrier protein

6.4.1.2	ACCOA + ATP + CO2 <-> MALCOA + ADP + PI

100	sll0336	accD, ycf11, zfpA acetyl-CoA carboxylase carboxyl transferase subunit beta

6.4.1.2	ACCOA + ATP + CO2 -> MALCOA + ADP + PI

101	sll0728	accA	acetyl-CoA carboxylase carboxyl transferase subunit alpha

6.4.1.2	ACCOA + ATP + CO2 -> MALCOA + ADP + PI

102	slr1556	ddh	2-hydroxyaciddehydrogenase	1.1.1.28	LAC + NAD <-> PYR + NADH

103	sll1019	gloB	glyoxalase II	3.1.2.6	LGT -> RGT + LAC

104	slr0091	lacl2	aldehyde dehydrogenase	1.2.1.3	LACAL + NADH -> PROP12D + NAD

105	sll0990	lacl3	formaldehyde dehydrogenase (glutathione) / alcohol dehydogenase

1.2.1.1	1.1.1.1	LACAL + RGT + NAD <-> LGT + NADH

106	slr0721	me	malic enzyme	1.1.1.38	MAL + NAD <-> CO2 + NADH + PYR

107	sll0920	ppc	phosphoenolpyruvate carboxylase	4.1.1.31	PEP + CO2 -> OA + PI

108	slr0301	ppsA	phosphoenolpyruvate synthase	2.7.9.2	PYR + ATP -> PEP + AMP + PI

109	sll1564	leuA	2-isopropylmalate synthase	2.3.3.13	OMVAL + ACCOA -> COA + IPPMAL

110	slr0186	leuA	2-isopropylmalate synthase	2.3.3.13	OMVAL + ACCOA -> COA + IPPMAL

Glyoxylate and dicarboxylate metabolism

111	sll0404	glcD	glycolate oxidase subunit GlcD	1.1.3.15	GLYCOLATE + O2 -> GLX + H2O2

112	sll1349	cbbZp	phosphoglycolate phosphatase	3.1.3.18	2PGLYCOL -> GLYCOLATE + PI

113	sll0107	eda, hga	2-dehydro-3-deoxyphosphogluconate aldolase

4.1.2.14	2KD6PG -> T3P1 + PYR

114	sll0107	edd	Phosphogluconate dehydratase	4.2.1.12	D6PGC -> 2KD6PG

115	sll0107	eda, hga	4-hydroxy-2-oxoglutarate aldolase	4.1.3.16	HOGLR <-> GLX + PYR

116	sll0753	folD	methylenetetrahydrofolate dehydrogenase (NADP+)

1.5.1.5	METHF + NADPH -> METTHF + NADP

117	sll0753	folD	methenyltetrahydrofolate cyclohydrolase

3.5.4.9	METHF <-> FTHF

118	sll0070	purU, tgs	formyltetrahydrofolate deformylase

3.5.1.10	FTHF -> FOR + THF

Carbon fixation

119	slr0009	rbcL	ribulose-bisphosphate carboxylase large chain

4.1.1.39	RIBBIP + O2 -> 2 3PG

120	slr0012	rbcS	ribulose-bisphosphate carboxylase small chain

4.1.1.39	RIBBIP + CO2 -> 3PG + 2PGLYCOL

121	slr0394	pgk	phosphoglycerate kinase	2.7.2.3	3PG + ATP <-> 13PDG + ADP

122	slr0394	pgk	phosphoglycerate kinase	2.7.2.3	13PDG + NADPH <-> T3P1 + NADP

123	0.167 T3P1 -> 0.167 F6P

124	0.833 T3P1 -> 0.833 RL5P

125	sll0018	cbbA, cfxA, fbaA, fda	fructose-bisphosphate aldolase class II

4.1.2.13	S17P <-> T3P2 + E4P

126	slr0943	fda	fructose-bisphosphate aldolase class I

4.1.2.13	S17P <-> T3P2 + E4P

127	slr0952	fbpII, fbp, fdp	fructose-16-bisphosphatase

3.1.3.11	S17P -> S7P + PI

128	slr2094	fbpI, glpX	fructose-16-/sedoheptulose-17-bisphosphatase GlpX protein

3.1.3.11	3.1.3.37	S17P -> S7P + PI

129	sll1525	prk, ptk	phosphoribulokinase	2.7.1.19	ATP + RL5P -> ADP + RIBBIP + PI

130	sll0402	aspC, aat aspartate aminotransferase	2.6.1.1	ASP + AKG <-> OA + GLU

131	sll0480	aspC, aat putative aminotransferase	2.6.1.1	ASP + AKG <-> OA + GLU

132	slr0036	aspC, aat aspartate aminotransferase	2.6.1.1	ASP + AKG <-> OA + GLU

C5-Branched dibasic acid metabolism

133	slr0370	gabD	succinate-semialdehyde dehydrogenase (NADP+)

1.2.1.16	SUCCSAL + NAD -> SUCC + NADH

134	slr0370	gabD	succinate-semialdehyde dehydrogenase (NADP+)

1.2.1.16	SUCCSAL + NADP -> SUCC + NADPH

135	sll1641	gad	glutamate decarboxylase	4.1.1.15	GLU -> GABA + CO2

136	sll1981	ilvB	acetolactate synthase large subunit	2.2.1.6	TPP + PYR -> 2AHTPP + CO2

137	slr2088	ilvG	acetolactate synthase large subunit	2.2.1.6	2AHTPP + PYR -> ACLAC + TPP

138	sll0065	ilvN	acetolactate synthase small subunit	2.2.1.6	2AHTPP + PYR -> ACLAC + TPP

139	sll1981	ilvB	acetolactate synthase large subunit	2.2.1.6	2 PYR -> ACLAC + CO2

140	slr2088	ilvG	acetolactate synthase large subunit	2.2.1.6	2 PYR -> ACLAC + CO2

141	sll0065	ilvN	acetolactate synthase small subunit	2.2.1.6	2 PYR -> ACLAC + CO2

Oxidative phosphorylation

142	slr1279	ndhC	NADH dehydrogenase I chain A	1.6.5.3	UBIQ + NADH -> UBIQOL + NAD + 3 HEXT

143	slr1280	ndhK, psbG1 NADH dehydrogenase I chain B

1.6.5.3	UBIQ + NADH -> UBIQOL + NAD + 3 HEXT

144	slr1281	ndhJ	NADH dehydrogenase I chain C	1.6.5.3	UBIQ + NADH -> UBIQOL + NAD + 3 HEXT

145	slr0261	ndhH	NADH dehydrogenase I chain D	1.6.5.3	UBIQ + NADH -> UBIQOL + NAD + 3 HEXT

146	sll1220	hoxE	diaphorase subunit of the bidirectional hydrogenase

1.6.5.3	UBIQ + NADH -> UBIQOL + NAD + 3 HEXT

147	sll1221	hoxF	diaphorase subunit of the bidirectional hydrogenase

1.6.5.3	UBIQ + NADH -> UBIQOL + NAD + 3 HEXT

148	sll1223	hoxU	diaphorase subunit of the bidirectional hydrogenase	1.6.5.3	UBIQ + NADH -> UBIQOL + NAD + 3 HEXT

149	sll0519	ndhA	NADH dehydrogenase I chain H	1.6.5.3	UBIQ + NADH -> UBIQOL + NAD + 3 HEXT

150	sll0520	ndhI	NADH dehydrogenase I chain I	1.6.5.3	UBIQ + NADH -> UBIQOL + NAD + 3 HEXT

151	sll0521	ndhG	NADH dehydrogenase I chain J	1.6.5.3	UBIQ + NADH -> UBIQOL + NAD + 3 HEXT

152	sll0522	ndhE	NADH dehydrogenase I chain K	1.6.5.3	UBIQ + NADH -> UBIQOL + NAD + 3 HEXT

153	sll0026	ndhF4	NADH dehydrogenase I chain L, involved in constitutive low-affinity CO2 uptake

1.6.5.3	UBIQ + NADH -> UBIQOL + NAD + 3 HEXT

154	sll1732	ndhF3	NADH dehydrogenase I chain L, involved in inducible high-affinity CO2 uptake

1.6.5.3	UBIQ + NADH -> UBIQOL + NAD + 3 HEXT

155	slr0844	ndhF1	NADH dehydrogenase I chain L, involved in photosystem-1 cyclic electron flow

1.6.5.3	UBIQ + NADH -> UBIQOL + NAD + 3 HEXT

156	sll0027	ndhD4	NADH dehydrogenase I chain M, involved in constitutive low-affinity CO2 uptake

1.6.5.3	UBIQ + NADH -> UBIQOL + NAD + 3 HEXT

157	sll1733	ndhD3	NADH dehydrogenase I chain M, involved in inducible high-affinity CO2 uptake

1.6.5.3	UBIQ + NADH -> UBIQOL + NAD + 3 HEXT

158	slr0331	ndhD1	NADH dehydrogenase I chain M, involved in photosystem-1 cyclic electron flow

1.6.5.3	UBIQ + NADH -> UBIQOL + NAD + 3 HEXT

159	slr1291	ndhD2	NADH dehydrogenase I chain M, involved in photosystem-1 cyclic electron flow

1.6.5.3	UBIQ + NADH -> UBIQOL + NAD + 3 HEXT

160	slr2007	ndhD5	NADH dehydrogenase I chain M	1.6.5.3	UBIQ + NADH -> UBIQOL + NAD + 3 HEXT

161	slr2009	ndhD6, ndhF	NADH dehydrogenase I chain M

1.6.5.3	UBIQ + NADH -> UBIQOL + NAD + 3 HEXT

162	sll0223	ndhB	NADH dehydrogenase I chain N	1.6.5.3	UBIQ + NADH -> UBIQOL + NAD + 3 HEXT

163	sll1484	ndbC, ndh	type II NADH dehydrogenase

1.6.99.3	UBIQ + NADH -> UBIQOL + NAD + 3 HEXT

164	slr0851	ndbA, ndh	type 2 NADH dehydrogenase

1.6.99.3	UBIQ + NADH -> UBIQOL + NAD + 3 HEXT

165	slr1743	ndbB, ndh	type 2 NADH dehydrogenase

1.6.99.3	UBIQ + NADH -> UBIQOL + NAD + 3 HEXT

166	sll0823	sdhB	succinate dehydrogenase iron-suflur protein

1.3.99.1	SUCC + FAD -> FADH2 + FUM

167	sll1625	sdhB	succinate dehydrogenase iron-suflur protein

1.3.99.1	UBIQ + FADH2 <-> UBIQOL + FAD

168	slr1233	frdA	succinate dehydrogenase flavoprotein subunit

1.3.99.1	UBIQ + FADH2 <-> UBIQOL + FAD

169	slr1138	ctaEI	cytochrome c oxidase subunit III	1.9.3.1	O2 + 4 FERO -> 4 FERI + 4 HEXT

170	slr2083	ctaE	cytochrome c oxidase subunit III	1.9.3.1	O2 + 4 FERO -> 4 FERI + 4 HEXT

171	slr1137	ctaDI, ctaD, coxI, coxA	cytochrome c oxidase subunit I

1.9.3.1	O2 + 4 FERO -> 4 FERI + 4 HEXT

172	slr2082	ctaD	cytochrome c oxidase subunit I	1.9.3.1	O2 + 4 FERO -> 4 FERI + 4 HEXT

173	sll0813	ctaC, coxB cytochrome c oxidase subunit II	1.9.3.1	O2 + 4 FERO -> 4 FERI + 4 HEXT

174	slr1136	ctaCI, ctaC, coxB	cytochrome c oxidase subunit II

1.9.3.1	O2 + 4 FERO -> 4 FERI + 4 HEXT

ATP synthesis

175	slr1622	ppa	inorganic pyrophosphatase	3.6.1.1	PPI -> 2 PI

176	sll0290	ppk	polyphosphate kinase	2.7.4.1	PPP + ADP <-> PPI + ATP

177	sll1322	atpI	ATP synthase A chain of CF(0)	3.6.3.14	ATP <-> ADP + PI + 3 HEXT

178	sll1323	atpG	ATP synthase subunit b' of CF(0)	3.6.3.14	ATP <-> ADP + PI + 3 HEXT

179	sll1324	atpF	ATP synthase B chain (subunit I) of CF(0)

3.6.3.14	ATP <-> ADP + PI + 3 HEXT

180	sll1325	atpD, atpH	ATP synthase delta chain of CF(1)

3.6.3.14	ATP <-> ADP + PI + 3 HEXT

181	sll1326	atpA	ATP synthase alpha chain	3.6.3.14	ATP <-> ADP + PI + 3 HEXT

182	sll1327	atpC, atpG	ATP synthase gamma chain

3.6.3.14	ATP <-> ADP + PI + 3 HEXT

183	slr1329	atpB, atpD	ATP synthase beta chain of CF(1)

3.6.3.14	ATP <-> ADP + PI + 3 HEXT

184	slr1330	atpE, atpC	ATP synthase epsilon chain of CF(1)

3.6.3.4	ATP <-> ADP + PI + 3 HEXT

185	ssl2615	atpH, atpE	ATP synthase C chain of CF(0)

3.6.3.4	ATP <-> ADP + PI + 3 HEXT

186	slr1330	atpE, atpC	ATP synthase epsilon chain of CF(1)

3.6.3.14	ATP <-> ADP + PI + 3 HEXT

187	slr1329	atpB, atpD	ATP synthase beta chain of CF(1)

3.6.3.14	ATP <-> ADP + PI + 3 HEXT

188	sll1327	atpC, atpG	ATP synthase gamma chain

3.6.3.14	ATP <-> ADP + PI + 3 HEXT

189	sll1326	atpA	ATP synthase alpha chain	3.6.3.14	ATP <-> ADP + PI + 3 HEXT

190	sll1325	atpD, atpH	ATP synthase delta chain of CF(1)

3.6.3.14	ATP <-> ADP + PI + 3 HEXT

191	sll1323	atpG	ATP synthase subunit b' of CF(0)	3.6.3.14	ATP <-> ADP + PI + 3 HEXT

192	sll1324	atpF	ATP synthase B chain (subunit I) of CF(0)

3.6.3.14	ATP <-> ADP + PI + 3 HEXT

193	ssl2615	atpH, atpE	ATP synthase C chain of CF(0)

3.6.3.14	ATP <-> ADP + PI + 3 HEXT

194	sll1322	atpI, atpB	ATP synthase A chain of CF(0)

3.6.3.14	ATP <-> ADP + PI + 3 HEXT

195	sll1321	ATP synthase protein I	3.6.3.14	ATP <-> ADP + PI + 3 HEXT

Photosynthesis

196	sll1867	psbA3	photosystem II D1 protein	4 UBIQ + 2 PLASTONE -> 2 PLASTOL + O2 + 8 HEXT

197	slr1181	psbA1	photosystem II D1 protein	5 UBIQ + 2 PLASTONE -> 2 PLASTOL + O2 + 8 HEXT

198	slr1311	psbA2	photosystem II D1 protein	6 UBIQ + 2 PLASTONE -> 2 PLASTOL + O2 + 8 HEXT

199	slr0906	psbB	photosystem II core light harvesting protein	7 UBIQ + 2 PLASTONE -> 2 PLASTOL + O2 + 8 HEXT

200	sll0851	psbC	photosystem II 44kDa reaction center protein	8 UBIQ + 2 PLASTONE -> 2 PLASTOL + O2 + 8 HEXT

201	sll0849	psbD1	photosystem II reaction center D2 protein	9 UBIQ + 2 PLASTONE -> 2 PLASTOL + O2 + 8 HEXT

202	slr0927	psbD2	photosystem II reaction center D2 protein	10 UBIQ + 2 PLASTONE -> 2 PLASTOL + O2 + 8 HEXT

203	ssr3451	psbE	cytochrome b559 alpha chain	11 UBIQ + 2 PLASTONE -> 2 PLASTOL + O2 + 8 HEXT

204	smr0006	psbF	cytochrome b559 beta chain	12 UBIQ + 2 PLASTONE -> 2 PLASTOL + O2 + 8 HEXT

205	ssl2598	psbH	photosystem II PsbH protein	13 UBIQ + 2 PLASTONE -> 2 PLASTOL + O2 + 8 HEXT

206	sml0001	psbI	photosystem II reaction center PsbI protein	14 UBIQ + 2 PLASTONE -> 2 PLASTOL + O2 + 8 HEXT

207	smr0008	psbJ	photosystem II PsbJ protein	15 UBIQ + 2 PLASTONE -> 2 PLASTOL + O2 + 8 HEXT

208	sml0005	psbK	photosystem II PsbK protein	16 UBIQ + 2 PLASTONE -> 2 PLASTOL + O2 + 8 HEXT

209	smr0007	psbL	photosystem II PsbL protein	17 UBIQ + 2 PLASTONE -> 2 PLASTOL + O2 + 8 HEXT

210	sml0003	psbM	photosystem II PsbM protein	18 UBIQ + 2 PLASTONE -> 2 PLASTOL + O2 + 8 HEXT

211	smr0009	psbN	photosystem II PsbN protein	19 UBIQ + 2 PLASTONE -> 2 PLASTOL + O2 + 8 HEXT

212	sll0427	psbO	photosystem II manganese-stabilizing polypeptide	20 UBIQ + 2 PLASTONE -> 2 PLASTOL + O2 + 8 HEXT

213	sll1418	psbP, psbP2	photosystem II oxygen-evolving complex 23kDa protein PsbP homolog

21 UBIQ + 2 PLASTONE -> 2 PLASTOL + O2 + 8 HEXT

214	smr0001	psbT, psbTc, ycf8	photosystem II PsbT protein	22 UBIQ + 2 PLASTONE -> 2 PLASTOL + O2 + 8 HEXT

215	sll1194	psbU	photosystem II 12kDa extrinsic protein	23 UBIQ + 2 PLASTONE -> 2 PLASTOL + O2 + 8 HEXT

216	sll0258	psbV	cytochrome c550	24 UBIQ + 2 PLASTONE -> 2 PLASTOL + O2 + 8 HEXT

217	sll1398	psb28, psbW, psb13, ycf79	photosystem II reaction center 13kDa protein

25 UBIQ + 2 PLASTONE -> 2 PLASTOL + O2 + 8 HEXT

218	slr1739	psb28	photosystem II 13kDa protein homolog formerly PsbW

26 UBIQ + 2 PLASTONE -> 2 PLASTOL + O2 + 8 HEXT

219	sml0002	psbX	photosystem II PsbX protein	27 UBIQ + 2 PLASTONE -> 2 PLASTOL + O2 + 8 HEXT

220	sml0007	psbY, ycf32	photosystem II protein Y	28 UBIQ + 2 PLASTONE -> 2 PLASTOL + O2 + 8 HEXT

221	slr1645	psb27, psbZ	photosystem II 11kDa protein	29 UBIQ + 2 PLASTONE -> 2 PLASTOL + O2 + 8 HEXT

222	slr1834	psaA	photosystem I P700 chlorophyll a apoprotein subunit Ia

FERDOXR + PLASTCYOX -> FERDOXO + PLASTCYRED + HEXT

223	slr1835	psaB	photosystem I P700 chlorophyll a apoprotein subunit Ib

FERDOXR + PLASTCYOX -> FERDOXO + PLASTCYRED + HEXT

224	ssl0563	psaC	photosystem I iron-sulfur center subunit VII	UBIQ + PLASTCYRED + FERDOXO -> FERDOXR + PLASTCYOX

225	slr0737	psaD	photosystem I reaction center subunit II	UBIQ + PLASTCYRED + FERDOXO -> FERDOXR + PLASTCYOX

226	ssr2831	psaE	photosystem I subunit IV	UBIQ + PLASTCYRED + FERDOXO -> FERDOXR + PLASTCYOX

227	sll0819	psaF	photosystem I reaction center subunit III precursor (PSI-F)

UBIQ + PLASTCYRED + FERDOXO -> FERDOXR + PLASTCYOX

228	smr0004	psaI	photosystem I reaction center subunit VIII	UBIQ + PLASTCYRED + FERDOXO -> FERDOXR + PLASTCYOX

229	sml0008	psaJ	photosystem I reaction center subunit IX	UBIQ + PLASTCYRED + FERDOXO -> FERDOXR + PLASTCYOX

230	sll0629	psaK	alternative photosystem I reaction center subunit X	UBIQ + PLASTCYRED + FERDOXO -> FERDOXR + PLASTCYOX

231	ssr0390	psaK2	alternative photosystem I reaction center subunit X	UBIQ + PLASTCYRED + FERDOXO -> FERDOXR + PLASTCYOX

232	slr1655	psaL	photosystem I reaction center subunit XI	UBIQ + PLASTCYRED + FERDOXO -> FERDOXR + PLASTCYOX

233	smr0005	psaM	photosystem I reaction center subunit XII	UBIQ + PLASTCYRED + FERDOXO -> FERDOXR + PLASTCYOX

234	sll1317	petA	apocytochrome f component of cytochrome b6/f complex

UBIQ + PLASTCYRED + FERDOXO -> FERDOXR + PLASTCYOX

235	slr0342	petB	cytochrome b6	PLASTOL + 2 PLASTCYOX -> PLASTONE + 2 PLASTCYRED

236	sll1182	petC3	cytochrome b6-f complex alternative iron-sulfur subunit (Rieske iron-sulfur protein)

1.10.99.1	PLASTOL + 2 PLASTCYOX -> PLASTONE + 2 PLASTCYRED

237	sll1316	petC, petC1	cytochrome b6-f complex iron-sulfur subunit (Rieske iron-sulfur protein)

1.10.99.1	PLASTOL + 2 PLASTCYOX -> PLASTONE + 2 PLASTCYRED

238	slr1185	petC2	cytochrome b6-f complex alternative iron-sulfur subunit (Rieske iron-sulfur protein)

1.10.99.1	PLASTOL + 2 PLASTCYOX -> PLASTONE + 2 PLASTCYRED

239	slr0343	petD	cytochrome b6-f complex subunit PetD	PLASTOL + 2 PLASTCYOX -> PLASTONE + 2 PLASTCYRED

240	sll0199	petE	plastocyanin	PLASTOL + 2 PLASTCYOX -> PLASTONE + 2 PLASTCYRED

241	smr0010	petG	cytochrome b6-f complex subunit V (PetG)	PLASTOL + 2 PLASTCYOX -> PLASTONE + 2 PLASTCYRED

242	ssl3803	cytochrome b6-f complex subunit 6	PLASTOL + 2 PLASTCYOX -> PLASTONE + 2 PLASTCYRED

243	smr0003	petM	cytochrome b6-f complex subunit PetM	PLASTOL + 2 PLASTCYOX -> PLASTONE + 2 PLASTCYRED

244	sml0004	petN	cytochrome b6-f complex subunit VIII	PLASTOL + 2 PLASTCYOX -> PLASTONE + 2 PLASTCYRED

245	sll1578	cpcA	phycocyanin alpha subunit	PLASTOL + 2 PLASTCYOX -> PLASTONE + 2 PLASTCYRED

246	sll1577	cpcB	phycocyanin beta subunit	PLASTOL + 2 PLASTCYOX -> PLASTONE + 2 PLASTCYRED

247	sll1579	cpcC2	phycobilisome rod linker polypeptide	PLASTOL + 2 PLASTCYOX -> PLASTONE + 2 PLASTCYRED

248	sll1580	cpcC1	phycobilisome rod linker polypeptide	PLASTOL + 2 PLASTCYOX -> PLASTONE + 2 PLASTCYRED

249	ssl3093	cpcD	phycobilisome small rod linker polypeptide	PLASTOL + 2 PLASTCYOX -> PLASTONE + 2 PLASTCYRED

250	slr1878	cpcE	phycocyanin alpha-subunit phycocyanobilin lyase

4.-.-.-	PLASTOL + 2 PLASTCYOX -> PLASTONE + 2 PLASTCYRED

251	sll1051	cpcF	phycocyanin alpha-subunit phycocyanobilin lyase	PLASTOL + 2 PLASTCYOX -> PLASTONE + 2 PLASTCYRED

252	sll1471	cpcG2	phycobilisome rod-core linker polypeptide	PLASTOL + 2 PLASTCYOX -> PLASTONE + 2 PLASTCYRED

253	slr2051	cpcG1	phycobilisome rod-core linker polypeptide	PLASTOL + 2 PLASTCYOX -> PLASTONE + 2 PLASTCYRED

254	slr2067	apcA	allophycocyanin alpha subunit	PLASTOL + 2 PLASTCYOX -> PLASTONE + 2 PLASTCYRED

255	slr1986	apcB	allophycocyanin beta subunit	PLASTOL + 2 PLASTCYOX -> PLASTONE + 2 PLASTCYRED

256	ssr3383	apcC	phycobilisome small core linker polypeptide	PLASTOL + 2 PLASTCYOX -> PLASTONE + 2 PLASTCYRED

257	sll0928	apcD	allophycocyanin-B	PLASTOL + 2 PLASTCYOX -> PLASTONE + 2 PLASTCYRED

258	slr0335	apcE	phycobilisome core-membrane linker polypeptide	PLASTOL + 2 PLASTCYOX -> PLASTONE + 2 PLASTCYRED

259	slr1459	apcF	phycobilisome core component	PLASTOL + 2 PLASTCYOX -> PLASTONE + 2 PLASTCYRED

260	sll1382	petF, fdx	ferredoxin petF-like protein expression is (-)regulated by by glucose

1.18.1.2	2 FERDOXR + NADP -> 2 FERDOXO + NADPH

261	slr0150	petF, fdx	ferredoxin petF-like protein expression is (-)regulated by by light

1.18.1.2	3 FERDOXR + NADP -> 2 FERDOXO + NADPH

262	slr1828	petF, fdx	ferredoxin petF-like protein no detectable expression

1.18.1.2	4 FERDOXR + NADP -> 2 FERDOXO + NADPH

263	ssl0020	petF, fed1	ferredoxin I essential for growth expression is induced by light

1.18.1.2	5 FERDOXR + NADP -> 2 FERDOXO + NADPH

264	sll0248	isiB	flavodoxin	1.18.1.2	6 FERDOXR + NADP -> 2 FERDOXO + NADPH

265	slr1643	petH	ferredoxin--NADP+ oxidoreductase1.18.1.2	7 FERDOXR + NADP -> 2 FERDOXO + NADPH

266	slr0899	cynS	cyanate lyase	4.2.1.104	CYAN + HCO3 <-> CARB + CO2

267	slr0051	ecaB, icfA	periplasmic beta-type carbonic anhydrase

4.2.1.1	CO2 -> H2CO3

268	slr1347	icfA	beta-type carbonic anhydrase localized in the carboxysome

4.2.1.1	CO2 -> H2CO3

Nitrogen metabolism

269	slr0898	nirA	ferredoxin-nitrite reductase	1.7.7.1	NITRITE + 6 FERDOXR -> NH3 + 6 FERDOXO

270	sll0573	arcC	carbamate kinase	2.7.2.2	ATP + CARB -> ADP + CAP

271	sll0171	gcvT	aminomethyltransferase	2.1.2.10	GLY + THF + NAD <-> METTHF + NADH + NH3 + CO2

272	sll0422	asp2	asparaginase	3.5.1.1	ASN -> NH3 + ASP

273	slr0710	gdhA	NADP-specific glutamate dehydrogenase

1.4.1.4	GLU + NADP <-> AKG + NH3 + NADPH

274	slr0710	gdhA	NADP-specific glutamate dehydrogenase

1.4.1.4	GLU + NAD <-> AKG + NH3 + NADH

275	slr0288	glnN	glutamate-ammonia ligase glutamine synthetase type III

6.3.1.2	GLU + ATP + NH3 -> ADP + PI + GLN

276	slr1756	glnA	glutamate-ammonia ligase glutamine synthetase type I

6.3.1.2	GLU + ATP + NH3 -> ADP + PI + GLN

277	slr2079	glt	glutaminase	3.5.1.2	GLN -> GLU + NH3

278	sll1502	gltB	NADH-dependent glutamate synthase large subunit

1.4.1.14	GLN + AKG + NADH -> 2 GLU + NAD

279	sll1027	gltD	NADH-dependent glutamate synthase small subunit

1.4.1.14	GLN + AKG + NADH -> 2 GLU + NAD

280	sll1499	glsF,gltS	ferredoxin-dependent glutamate synthase

1.4.7.1	GLN + AKG + 2 FERDOXR -> 2 GLU + 2 FERDOXO

281	sll1454	narB	ferredoxin-nitrate reductase	1.7.7.2	NITRATE + 2 FERDOXR -> NITRITE + 2 FERDOXO

Sulfur metabolism

282	slr0676	cysC	adenylylsulfate 3-phosphotransferase

2.7.1.25	APS + ATP -> ADP + PAPS

283	slr1165	met3, sopT	sulfate adenylyltransferase	2.7.7.4	SLF + ATP -> PPI + APS

284	slr1791	cysH	phosphoadenosine phosphosulfate reductase

1.8.4.8	RTHIO + PAPS -> PAP + H2SO3 + OTHIO

285	slr0963	sir	ferredoxin-sulfite reductase	1.8.7.1	H2SO3 + 3 FERDOXR -> H2S + 3 FERDOXO

286	slr1348	cysE	serine acetyltransferase	2.3.1.30	SER + ACCOA -> ASER + COA

287	sll0712	cysM	cysteine synthase	2.5.1.47	ASER + H2S -> CYS + AC

288	slr1842	cysK	cysteine synthase	2.5.1.47	ASER + H2S -> CYS + AC

Fatty acid biosynthesis

289	sll0053	accC, fabG	biotin carboxylase	6.3.4.14	ACCOA + ATP + CO2 <-> MALCOA + ADP + PI

290	sll1069	fabF	3-oxoacyl-[acyl-carrier-protein] synthase

2.3.1.41	ACCOA + ACP <-> ACACP + COA

291	slr1332	fabJ	3-oxoacyl-[acyl-carrier-protein] synthase

2.3.1.41	MALACP + ACACP -> ACP + CO2 + 3OACP

292	slr1511	fabH	3-oxoacyl-[acyl-carrier-protein] synthase III

2.3.1.41	MALACP + ACACP -> ACP + CO2 + 3OACP

293	slr2023	fabD	malonyl CoA-acyl carrier protein transacylase

2.3.1.39	MALCOA + ACP <-> MALACP + COA

294	slr0886	fabG1	3-oxoacyl-[acyl-carrier protein] reductase 1

1.1.1.100	ACCOA + ACP <-> ACACP + COA

295	slr1994	fabG2	3-oxoacyl-[acyl-carrier protein] reductase 2

1.1.1.100	ACCOA + ACP <-> ACACP + COA

296	slr1510	plsX	fatty acid/phospholipid synthesis protein

GL3P +0.035 C140ACP +0.102 C141ACP +0.717 C160ACP +0.142 C161ACP +1.004 C181ACP -> 2 ACP + PA

297	slr1993	fas1	b-Ketoacyl-ACP synthase (C12,0), fatty acyl CoA synthase

1.3.1.9	ACACP + 5 MALACP + 10 NADPH -> 10 NADP + C120ACP + 5 CO2 + 5 ACP

298	slr1609	FAS2	b-Ketoacyl-ACP synthase (C14,0)	1.3.1.9	ACACP + 6 MALACP + 12 NADPH -> 12 NADP + C140ACP + 6 CO2 + 6 ACP

299	sll0990	FAS2	b-Ketoacyl-ACP synthase I (C14,1)	1.3.1.9	ACACP + 6 MALACP + 11 NADPH -> 11 NADP + C141ACP + 6 CO2 + 6 ACP

300	slr0091	FAS2	b-Ketoacyl-ACP synthase I (C16,0)	1.3.1.9	ACACP + 7 MALACP + 14 NADPH -> 14 NADP + C160ACP + 7 CO2 + 7 ACP

301	FAS2	b-Ketoacyl-ACP synthase I (C16,1)	1.3.1.9	ACACP + 7 MALACP + 13 NADPH -> 13 NADP + C161ACP + 7 CO2 + 7 ACP

302	FAS2	b-Ketoacyl-ACP synthase I (C18,1)	1.3.1.14	ACACP + 8 MALACP + 15 NADPH -> 15 NADP + C181ACP + 8 CO2 + 8 ACP

Sterol biosynthesis

303	sll1945	dxs	probable 1-deoxyxylulose-5-phosphate synthase

2.2.1.7	PYR + T3P1 -> 1DX5P + CO2

304	sll0019	dxr1	1-deoxy-D-xylulose 5-phosphate reductoisomerase

1.1.1.267	1DX5P + NADPH -> 2CMDE4P + NADP

305	sll0019	dxr2	1-deoxy-D-xylulose 5-phosphate reductoisomerase

1.1.1.267	1DX5P + NADH -> 2CMDE4P + NAD

306	slr0951	dip	4-diphosphocytidyl-2C-methyl-D-erythritol synthase

2.7.7.60	2CMDE4P + CTP -> 4C2ME + PPI

307	sll1556	iso	isopentenyl-diphosphate delta-isomerase

5.3.3.2	IPPP <-> DMPP

308	slr0739	crtE	geranylgeranyl pyrophosphate synthase

2.5.1.29	DMPP + IPPP -> GPP + PPI

309	slr1255	crtB	phytoene synthase	2.5.1.32	GPP + IPPP -> FPP + PPI

310	slr1254	crtP, pds, crtD	phytoene dehydrogenase, phytoene desaturase

1.3.99.-	5 IPPP + FPP -> OPP + 5 PPI

311	slr0940	crtQ-2, crtQ, zds	zeta-carotene desaturase	1.14.99.30	8 IPPP + FPP -> UDPP + 8 PPI

Purine metabolism

312	slr1054	adp	ADP-ribose pyrophosphatase	3.6.1.13	RIB -> R5P + AMP

313	sll0757	purF	amidophosphoribosyltransferase	2.4.2.14	PRPP + GLN -> PPI + GLU + PRAM

314	slr1159	purD	phosphoribosylamine--glycine ligase

6.3.4.13	PRAM + ATP + GLY <-> ADP + PI + GAR

315	slr0477	purN	phosphoribosylglycinamide formyltransferase

2.1.2.2	GAR + FTHF -> THF + FGAR

316	sll1056	purL	phosphoribosylformyl glycinamidine synthetase II

6.3.5.3	FGAR + ATP + GLN -> GLU + ADP + PI + FGAM

317	slr0520	purL2	phosphoribosyl formylglycinamidine synthase

6.3.5.3	FGAR + ATP + GLN -> GLU + ADP + PI + FGAM

318	slr0838	purM, purG	phosphoribosyl formylglycinamidine cyclo-ligase

6.3.3.1	FGAM + ATP -> ADP + PI + AIR

319	sll0901	purE, ade6	phosphoribosylaminoimidazole carboxylase catalytic subunit

4.1.1.21	CAIR <-> AIR + CO2

320	sll0578	purK	phosphoribosylaminoimidazole carboxylase ATPase subunit

4.1.1.21	CAIR <-> AIR + CO2

321	slr1226	purC	phosphoribosyl aminoidazole succinocarboxamide synthetase

6.3.2.6	CAIR + ATP + ASP <-> ADP + PI + SAICAR

322	sll0421	purB	adenylosuccinate lyase	4.3.2.2	SAICAR <-> FUM + AICAR

323	sll0421	purB	adenylosuccinate lyase	4.3.2.2	ASUC <-> FUM + AMP

324	slr0597	purH	phosphoribosylaminoimidazolecarboxamide formyltransferase / IMP cyclohydrolase

2.1.2.3	3.5.4.10	AICAR + FTHF <-> THF + PRFICA

325	slr0597	purH	phosphoribosylaminoimidazolecarboxamide formyltransferase / IMP cyclohydrolase

2.1.2.3	3.5.4.10	PRFICA <-> IMP

326	slr1722	guaB, gnaB	IMP dehydrogenase subunit	1.1.1.205	IMP + NAD -> NADH + XMP

327	slr0213	guaA	GMP synthase (glutamine-hydrolysing)

6.3.5.2	XMP + ATP + GLN -> GLU + AMP + PPI + GMP

328	slr1123	gmk, spoR	guanylate kinase	2.7.4.8	GMP + ATP <-> GDP + ADP

329	slr1164	nrdA	ribonucleoside-diphosphate reductase alpha chain

1.17.4.1	GDP + RTHIO -> DGDP + OTHIO

330	slr0591	nrdF	ribonucleoside-diphosphate reductase beta chain

1.17.4.1	ADP + RTHIO -> DADP + OTHIO

331	sll1430	apt	adenine phosphoribosyltransferase	2.4.2.7	GN + PRPP -> PPI + GMP

332	sll1430	apt	adenine phosphoribosyltransferase	2.4.2.7	AD + PRPP -> PPI + AMP

333	sll1059	adk	adenylate kinase	2.7.4.3	ATP + AMP <-> 2 ADP

334	sll1815	adk	adenylate kinase	2.7.4.3	ATP + DAMP <-> ADP + DADP

335	sll1852	ndkR	nucleoside diphosphate kinase	2.7.4.6	GDP + ATP <-> GTP + ADP

336	sll1852	ndk2	nucleoside diphosphate kinase	2.7.4.6	DGDP + ATP <-> DGTP + ADP

337	sll1852	ndk3	nucleoside diphosphate kinase	2.7.4.6	ATP + IDP <-> ADP + ITP

338	sll1852	ndk4	nucleoside diphosphate kinase	2.7.4.6	ATP + DADP <-> ADP + DATP

339	sll1852	ndk5	nucleoside diphosphate kinase	2.7.4.6	ATP + DIDP <-> ADP + DITP

340	sll0398	dgt	dGTP triphosphohydrolase	3.1.5.1	DGTP -> DG + PPP

341	sll1750	ureC	urease alpha subunit	3.5.1.5	UREA -> CO2 + 2 NH3

342	sll0420	ureB	urease beta subunit	3.5.1.5	UREA -> CO2 + 2 NH3

343	slr1256	ureA	urease gamma subunit	3.5.1.5	UREA -> CO2 + 2 NH3

344	sll1161	cya3	putative adenylate cyclase	4.6.1.1	ATP -> cAMP + PPI

345	slr1991	cya1	adenylate cyclase	4.6.1.1	GTP -> cGMP + PPI

346	sll0646	cya2	guanylate cyclase	4.6.1.2	GTP -> cGMP + PPI

347	sll0646	cya2	guanylate cyclase	4.6.1.2	ITP -> cIMP + PPI

348	sll1823	purA	adenylosuccinate synthetase	6.3.4.4	IMP + GTP + ASP -> GDP + PI + ASUC

349	sll0573	arcC	carbamate kinase	2.7.2.2	ATP + NH3 + CO2 -> ADP + CAP

Pyrimidine metabolism

350	sll0370	carB, pyrA	carbamoyl-phosphate synthase large chain

6.3.5.5	GLN + 2 ATP + CO2 -> GLU + CAP + 2 ADP + PI

351	sll1498	carA, pyrA	carbamoyl-phosphate synthase small chain

6.3.5.5	GLN + 2 ATP + CO2 -> GLU + CAP + 2 ADP + PI

352	slr1476	pyrB	aspartate carbamoyltransferase catalytic chain

2.1.3.2	CAP + ASP -> CAASP + PI

353	sll1018	pyrC	dihydroorotase	3.5.2.3	CAASP <-> DOROA

354	slr0406	pyrC	dihydroorotase	3.5.2.3	CAASP <-> DOROA

355	slr1418	pyrD	dihydroorotate oxidase	1.3.3.1	DOROA + O2 <-> H2O2 + OROA

356	slr0185	umpS	orotate phosphoribosyltransferase	2.4.2.10	OROA + PRPP <-> PPI + OMP

357	sll0838	pyrF	orotidine 5'-phosphate decarboxylase

4.1.1.23	OMP -> CO2 + UMP

358	sll1249	kcy	pantoate--beta-alanine ligase / cytidylate kinase

6.3.2.1	2.7.4.14	CMP + ATP <-> ADP + CDP

359	sll1249	panC	pantoate--beta-alanine ligase / cytidylate kinase

6.3.2.1	2.7.4.14	UMP + ATP <-> ADP + UDP

360	sll1852	ndk	nucleoside diphosphate kinase	2.7.4.6	ATP + UDP <-> ADP + UTP

361	sll1852	ndk	nucleoside diphosphate kinase	2.7.4.6	ATP + CDP <-> ADP + CTP

362	sll1852	ndk	nucleoside diphosphate kinase	2.7.4.6	ATP + DTDP <-> ADP + DTTP

363	sll1852	ndkr	nucleoside diphosphate kinase	2.7.4.6	ATP + DCDP <-> ADP + DCTP

364	sll1852	ndkr	nucleoside diphosphate kinase	2.7.4.6	ATP + DUDP <-> ADP + DUTP

365	sll1443	pyrG	CTP synthase	6.3.4.2	UTP + GLN + ATP -> GLU + CTP + ADP + PI

366	sll1443	pyrG	CTP synthase	6.3.4.2	ATP + UTP + NH3 -> ADP + PI + CTP

367	sll1258	dcd	deoxycytidine triphosphate deaminase

3.5.4.13	DCTP -> DUTP + NH3

368	sll1258	dcd	deoxycytidine triphosphate deaminase

3.5.4.13	CTP -> UTP + NH3

369	sll0368	pyrR	pyrimidine operon regulatory protein PyrR

2.4.2.9	URA + PRPP -> UMP + PPI

370	sll1035	upp, uraP	uracil phosphoribosyltransferase	2.4.2.9	URA + PRPP -> UMP + PPI

371	slr1237	codA	cytosine deaminase	3.5.4.1	CYTS -> URA + NH3

372	slr0600	nrdc	putative thioredoxin reductase	1.8.1.9	OTHIO + NADPH -> NADP + RTHIO

373	slr1164	nrdA	ribonucleoside-diphosphate reductase alpha chain

1.17.4.1	CDP + RTHIO -> DCDP + OTHIO

374	slr0591	nrdF	ribonucleoside-diphosphate reductase beta chain

1.17.4.1	UDP + RTHIO -> DUDP + OTHIO

375	slr0379	tmk	thymidylate kinase	2.7.4.9	DT + ATP -> ADP + DTMP

376	slr0379	tmk	thymidylate kinase	2.7.4.9	ATP + DTMP <-> ADP + DTDP

377	slr0379	tmk	thymidylate kinase	2.7.4.9	ATP + DUMP <-> ADP + DUDP

Nucleotide sugars metabolism

378	slr0983	rfbF	alpha-D-glucose-1-phosphate cytidylyltransferase

2.7.7.33	G1P + DTTP <-> DTDPGLUC + PPI

379	sll0207	rfbA	glucose-1-phosphate thymidylyltransferase

2.7.7.24	G1P + DTTP <-> DTDPGLUC + PPI

380	slr0809	rfbB	dTDP-glucose 4-6-dehydratase	4.2.1.46	DTDPGLUC -> DTDP4D6DG

381	slr0836	rfbB	dTDP-glucose 4 6-dehydratase	4.2.1.46	DTDPGLUC -> DTDP4D6DG

382	slr0985	rfbC	dTDP-4-dehydrorhamnose 3 5-epimerase

5.1.3.13	DTDP4D6DG -> DTDP4D6DM

383	slr1933	rfbC	dTDP-4-dehydrorhamnose 3 5-epimerase

5.1.3.13	DTDP4D6DG -> GDP4DDMAN

384	sll1395	rfbD	dTDP-6-deoxy-L-mannose-dehydrogenase

1.1.1.133	GDP4DDMAN + NADPH -> GDP4DMAN + NADP

385	sll1395	rfbD	dTDP-6-deoxy-L-mannose-dehydrogenase

1.1.1.133	DTDP4D6DM + NADPH -> DTDP6DM + NADP

386	slr1067	galE	UDP-glucose 4-epimerase	5.1.3.2	DTDPGLUC <-> DTDPGLAC

387	slr1078	galE	UDP-glucose 4-epimerase	5.1.3.2	DTDPGLUC <-> DTDPGLAC

Glutamate metabolism

388	sll0179	gltX	glutamyl-tRNA synthetase	6.1.1.17	GLU + ATP -> GTRNA + AMP + PPI

389	sll0220	glmS	glucosamine--fructose-6-phosphate aminotransferase

2.6.1.16	F6P + GLN -> GLU + GA6P

390	slr1746	murI	glutamate racemase	5.1.1.3	GLU <-> DEXGLU

391	slr1238	gshB	glutathione synthetase	6.3.2.3	GLY + GC + ATP -> RGT + PI + ADP

392	slr1691	nadE	NH(3)-dependent NAD(+) synthetase

6.3.5.1	NADH + ATP + GLN -> NAD + AMP + PPI + GLU

393	slr0213	guaA	GMP synthase (glutamine-hydrolysing)

6.3.5.2	XMP + ATP + NH3 -> AMP + PPI + GMP

Alanine and aspartate metabolism

394	sll0495	asnS	asparaginyl-tRNA synthetase	6.1.1.22	ATP + ASP + TRNA -> AMP + PPI + ASPTRNA

395	slr1133	argH	L-argininosuccinate lyase	4.3.2.1	ARGSUCC <-> FUM + ARG

396	slr0827	alr	alanine racemase	5.1.1.1	ALA <-> DEXALA

397	sll0892	panD	aspartate 1-decarboxylase	4.1.1.11	ASP -> CO2 + bALA

398	sll1641	gad	glutamate decarboxylase	4.1.1.15	ASP -> CO2 + bALA

399	slr1720	aspS	aspartyl-tRNA synthetase	6.1.1.12	ATP + ASP + TRNA -> AMP + PPI + ASPARTRNA

400	slr0585	argG	argininosuccinate synthase	6.3.4.5	CITR + ASP + ATP -> AMP + PPI + ARGSUCC

401	sll0631	nadB	L-aspartate oxidase	1.4.3.16	ASP + O2 -> OA + NH3 + H2O2

402	slr0657	lysC	aspartate kinase	2.7.2.4	ASP + ATP -> ADP + BASP

403	slr0549	asd	aspartate beta-semialdehyde dehydrogenese

1.2.1.11	BASP + NADPH -> NADP + PI + ASPSA

Glycine serine and threonine metabolism

404	sll0455	thrA	homoserine dehydrogenase	1.1.1.3	ASPSA + NADH <-> NAD + HSER

405	sll0456	thrA	homoserine dehydrogenase	1.1.1.4	ASPSA + NADH <-> NAD + HSER

Arginine and proline metabolism

406	slr0661	proC	pyrroline-5-carboxylate reductase	1.5.1.2	P5C + NADPH <-> PRO + NADP

407	slr0661	proC	pyrroline-5-carboxylate reductase	1.5.1.2	P5C + NADH <-> PRO + NAD

408	slr0661	proC	pyrroline-5-carboxylate reductase	1.5.1.2	PHC + NADPH -> HPRO + NADP

409	slr0661	proC	pyrroline-5-carboxylate reductase	1.5.1.2	PHC + NADH -> HPRO + NAD

410	sll1561	putA	delta-1-pyrroline-5-carboxylate dehydrogenase

1.5.1.12	1.5.99.8	GLUGSAL + NAD <-> NADH + GLU

411	sll1561	putA	delta-1-pyrroline-5-carboxylate dehydrogenase

1.5.1.12	1.5.99.8	P5C + NAD -> GLU + NADH

412	sll1561	putA	delta-1-pyrroline-5-carboxylate dehydrogenase

1.5.1.12	1.5.99.8	P5C + NADP -> GLU + NADPH

413	sll1561	putA	delta-1-pyrroline-5-carboxylate dehydrogenase

1.5.1.12	1.5.99.8	HPRO + FAD -> PHC + FADH2

414	sll1561	putA	delta-1-pyrroline-5-carboxylate dehydrogenase

1.5.1.12	1.5.99.8	PHC + NADH -> ERYHYGLU + NAD

415	sll1561	putA	delta-1-pyrroline-5-carboxylate dehydrogenase

1.5.1.12	1.5.99.8	PHC + NADPH -> ERYHYGLU + NADP

416	sll0402	aspE	aspartate aminotransferase	2.6.1.1	ERYHYGLU + AKG -> GLU + HYOXOGLUT

417	sll0480	aspF	putative aminotransferase	2.6.1.1	ERYHYGLU + AKG -> GLU + HYOXOGLUT

418	slr0036	aspG	aspartate aminotransferase	2.6.1.1	ERYHYGLU + AKG -> GLU + HYOXOGLUT

419	sll0107	eda	2-dehydro-3-deoxyphosphogluconate aldolase / 4-hydroxy-2-oxoglutarate aldolase

4.1.2.14	4.1.3.16	HYOXOGLUT <-> GLX + PYR

420	sll1425	proS	proline-tRNA ligase	6.1.1.15	ATP + PRO + TRNA -> AMP + PPI + PROTRNA

421	sll0502	argS	arginyl-tRNA synthetase	6.1.1.19	ATP + ARG + TRNA -> AMP + PPI + ALTRNA

422	slr0596	cre	creatinine amidohydrolase	3.5.2.10	CREAT <-> CRETNIN

423	slr0662	speA	arginine decarboxylase	4.1.1.19	ARG -> AGMAT + CO2

424	slr1312	speA	arginine decarboxylase	4.1.1.19	ARG -> AGMAT + CO2

425	sll0228	speB1	agmatinase	3.5.3.11	AGMAT -> PTRSC + UREA

426	sll1077	speB2	agmatine ureohydrolase	3.5.3.11	AGMAT -> PTRSC + UREA

427	sll0828	nylA	6-aminohexanoate-cyclic-dimer hydrolase

3.5.1.4	4GUANAM -> 4GUANBUT + NH3

428	slr0091	ald7	aldehyde dehydrogenase	1.2.1.3	4ACEBANAL + NAD -> 4ACEBATE + NADH

429	slr0091	ald8	aldehyde dehydrogenase	1.2.1.3	4AMBANAL + NAD -> GABA + NADH

430	slr0091	ald9	aldehyde dehydrogenase	1.2.1.3	4AMBANAL + NADP -> GABA + NADPH

431	sll0899	glmU	UDP-N-acetylglucosamine pyrophosphorylase / diamine N-acetyltransferase

2.7.7.23	2.3.1.57	ACCOA + PTRSC -> COA + APRUT

432	sll0456	hom	homoserine dehydrogenase	1.1.1.3	ASPSA + NADPH <-> NADP + HSER

433	sll1760	thrB	homoserine kinase	2.7.1.39	HSER + ATP -> ADP + PHSER

434	sll1172	thrC	threonine synthase	4.2.3.1	PHSER -> PI + THR

435	sll1688	thrC	threonine synthase	4.2.3.1	PHSER -> PI + THR

436	sll1931	glyA	serine hydroxymethyltransferase	2.1.2.1	THF + SER <-> GLY + METTHF

437	sll1908	serA	phosphoglycerate dehydrogenase	1.1.1.95	3PG + NAD -> NADH + PHP

438	slr0293	gcvP	glycine dehydrogenase	1.4.4.2	GLY + THF + NAD -> METTHF + NADH + CO2 + NH3

439	sll0171	gcvT	aminomethyltransferase	2.1.2.10	GLY + THF + NADP -> METTHF + NADPH + CO2 + NH3

440	slr0638	glyQ, glyS(a)	glycyl-tRNA synthetase alpha chain

6.1.1.14	ATP + GLY + TRNA -> AMP + PPI + GLYCTRNA

441	slr0220	glyS	glycyl-tRNA synthetase beta chain	6.1.1.14	ATP + GLY + TRNA -> AMP + PPI + GLYCTRNA

442	slr1703	serS	seryl-tRNA synthetase	6.1.1.11	ATP + SER + TRNA -> AMP + PPI + SERTRNA

443	slr2072	ilvA	L-threonine deaminase	4.3.1.19	SER <-> PYR + NH3

444	slr2072	ilvA	L-threonine deaminase	4.3.1.19	THR -> OBUT + NH3

445	sll0078	thrS	threonyl-tRNA synthetase	6.1.1.3	ATP + THR + TRNA -> AMP + PPI + THRTRNA

Methionine metabolism

446	slr0070	fmt	methionyl-tRNA formyltransferase	2.1.2.9	FTHF + MTRNA -> THF + FMRNA

447	sll0927	metX	S-adenosylmethionine synthetase	2.5.1.6	MET + ATP -> PPI + PI + SAM

448	slr0214	cytosine-specific	methyltransferase	4.4.1.-	OBUT + NH3 <-> HSER

449	slr0215	cytosine-specific	methyltransferase	2.3.1.29	2A3O + COA <-> ACCOA + GLY

450	slr0649	metS	methionyl-tRNA synthetase	6.1.1.10	ATP + MET + TRNA -> AMP + PPI + MTRNA

451	slr0212	metH	5-methyltetrahydrofolate-homocysteine methyltransferase

2.1.1.13	HCYS + MTHF -> MET + THF

Cysteine metabolism

452	slr0958	cysS	cysteinyl-tRNA synthetase	6.1.1.16	ATP + CYS + TRNA -> AMP + PPI + CYSTRNA

453	sll0712	cysM	cysteine synthase	2.5.1.47	ASER + THIOSULF -> SULFCYS + AC

454	slr1842	cysK	cysteine synthase	2.5.1.47	ASER + THIOSULF + RTHIO -> OTHIO + CYS + H2SO3 + AC

455	sll0402	"aspC, aat"	aspartate aminotransferase	2.6.1.1	MERCAPPYR + GLU -> CYS + AKG

456	cys3	cystathionine gamma-lyase	4.4.1.1	LLCT -> CYS + NH3 + OBUT

457	cyanom3	2.5.1.48	HSER + SUCCOA -> COA + OSLHSER

458	SAM4	PUTATIVE COBALAMIN-DEPENDENT HOMOCYSTEINE S-METHYLTRANSFERASE

2.1.1.37	SAM + HCYS -> SAH + MET

459	sll1234	ahcY	S-adenosylhomocysteine hydrolase	3.3.1.1	SAH <-> HCYS + ADENOS

460	CYS4	CYSTATHIONINE BETA-SYNTHASE

4.2.1.22	SER + HCYS -> LLCT

461	CYS5	CYSTATHIONINE GAMMA-LYASE

4.4.1.1	LLCT -> CYS + HSER

462	sll0480	cys6	putative aminotransferase	2.6.1.1	SULFOPYR + GLU <-> CYSATE + AKG

463	slr0036	aspC	aspartate aminotransferase	2.6.1.1	SULFALA + AKG -> SULFYPYR + GLU

Valine leucine and isoleucine degradation

464	slr0032	ilvE	branched-chain amino acid aminotransferase

2.6.1.42	AKG + LEU -> OICAP + GLU

465	slr0032	ilvE	branched-chain amino acid aminotransferase

2.6.1.42	AKG + VAL -> OMVAL + GLU

466	slr0032	ilvE	branched-chain amino acid aminotransferase

2.6.1.42	AKG + ILE -> OIOI + GLU

467	slr0229	mmsB	3-hydroxyisobutyrate dehydrogenase

1.1.1.31	HYDROXBUT + NAD <-> MALDEHYDE + NADH

468	slr0229	mmsB	3-hydroxyisobutyrate dehydrogenase

1.1.1.31	HYDROXBUT + NADP <-> MALDEHYDE + NADPH

469	slr0091	ald1	aldehyde dehydrogenase	1.2.1.3	MALDEHYDE + NAD -> MALNATE + NADH

470	slr0091	ald2	aldehyde dehydrogenase	1.2.1.3	MALDEHYDE + NADP -> MALNATE + NADPH

Valine leucine and isoleucine biosynthesis

471	slr2088	ilvG	acetolactate synthase large subunit	2.2.1.6	2AHTPP + OBUT -> ACEBUT + TPP

472	sll0065	ilvN	acetolactate synthase small subunit	2.2.1.6	2AHTPP + OBUT -> ACEBUT + TPP

473	sll1363	ilvC	ketol-acid reductoisomerase	1.1.1.86	ACLAC + NADPH -> DHMVA + NADP

474	sll1363	ilvC	ketol-acid reductoisomerase	1.1.1.86	ACEBUT + NADPH -> DHVAL + NADP

475	slr0452	ilvD	dihydroxyacid dehydratase	4.2.1.9	DHMVA -> OMVAL

476	slr0452	ilvD	dihydroxyacid dehydratase	4.2.1.9	DHVAL -> OXMET

477	slr0032	ilvE	branched-chain amino acid aminotransferase

2.6.1.42	OXMET + GLU -> AKG + ILE

478	slr0032	ilvE	branched-chain amino acid aminotransferase

2.6.1.42	OIOI + GLU -> AKG + ILE

479	slr0032	ilvE	branched-chain amino acid aminotransferase

2.6.1.42	OMVAL + GLU -> AKG + VAL

480	slr0032	ilvE	branched-chain amino acid aminotransferase

2.6.1.42	OICAP + GLU -> AKG + LEU

481	sll1470	leuC	3-isopropylmalate dehydratase large subunit

4.2.1.33	IPPMAL <-> IPPMAL3

482	sll1444	leuD	3-isopropylmalate dehydratase small subunit

4.2.1.33	IPPMAL <-> IPPMAL3

483	slr1517	leuB	3-isopropylmalate dehydrogenase	1.1.1.85	IPPMAL3 + NAD -> NADH + OICAP + CO2

484	sll1074	leuS	leucyl-tRNA synthetase	6.1.1.4	ATP + LEU + TRNA -> AMP + PPI + LUCETRNA

485	sll1362	ileS	isoleucyl-tRNA synthetase	6.1.1.5	ATP + ILE + TRNA -> AMP + PPI + ILETRNA

486	slr0557	valS	valyl-tRNA synthetase	6.1.1.9	ATP + VAL + TRNA -> AMP + PPI + VALTRNA

Lysine biosynthesis

487	slr0550	dapA	dihydrodipicolinate synthase	4.2.1.52	ASPSA + PYR -> D23PIC

488	sll1058	dapB	dihydrodipicolinate reductase	1.3.1.26	D23PIC + NADPH -> NADP + PIP26DX

489	sll0006	aspC	N-succinyldiaminopimelate aminotransferase

2.6.1.17	NS2A6O + GLU <-> AKG + NS26DP

490	sll0938	aspD	N-succinyldiaminopimelate aminotransferase

2.6.1.17	NS2A6O + GLU <-> AKG + NS26DP

491	slr1665	dapF	diaminopimelate epimerase	5.1.1.7	D26PIM <-> MDAP

492	sll0504	lysA	diaminopimelate decarboxylase	4.1.1.20	MDAP -> CO2 + LYS

493	slr1550	lysS	lysyl-tRNA synthetase	6.1.1.6	ATP + LYS + TRNA -> AMP + PPI + LLTRNA

494	slr0528	murE	UDP-MurNac-tripeptide synthetase	6.3.2.13	UALGLU + ATP + MDAP -> ADP + PI + UALGLUXXX

495	slr1351	murF, mra	UDP-N-acetylmuramoylalanyl-D-glutamyl-2 6-diaminopimelate-D-alanyl-D-alanine ligase

6.3.2.10	UALGLUXXX + ATP + ALAL -> ADP + PI + UALGLUYYY

Lysine degradation

496	sll1683	cad	lysine decarboxylase	4.1.1.18	LYS -> CO2 + CADV

497	sll1931	glyA	serine hydroxymethyltransferase	2.1.2.1	3H3N6MLYS -> GLY + 4TRIMBUT

498	slr0091	ald4	aldehyde dehydrogenase	1.2.1.3	4TRIMBUT + NAD -> 4TRIMBATE + NADH

499	sll0902	argF	ornithine carbamoyltransferase chain f

2.1.3.3	ORN + CAP -> CITR + PI

Histidine metabolism

500	sll0900	hisG	ATP phosphoribosyltransferase	2.4.2.17	PRPP + ATP -> PPI + PRBATP

501	slr0608	hisI	phosphoribosyl-AMP cyclohydrolase / phosphoribosyl-ATP pyrophosphohydrolase

3.5.4.19	3.6.1.31	PRBATP -> PPI + PRBAMP

502	slr0608	hisI	phosphoribosyl-AMP cyclohydrolase / phosphoribosyl-ATP pyrophosphohydrolase

3.5.4.19	3.6.1.31	PRBAMP -> PRFP

503	slr0652	hisA	phosphoribosylformimino-5-aminoimidazole carboxamide ribotide isomerase

5.3.1.16	PRFP -> PRLP

504	slr0500	hisB	imidazoleglycerol-phosphate dehydratase

4.2.1.19	DIMGP -> IMACP

505	sll1713	hisC	histidinol-phosphate aminotransferase

2.6.1.9	IMACP + GLU -> AKG + HISOLP

506	sll1958	hisD	histidinol phosphate aminotransferase

2.6.1.9	IMACP + GLU -> AKG + HISOLP

507	slr0682	hisE	histidinol dehydrogenase	1.1.1.23	HISOL + 2 NAD -> HIS + 2 NADH

508	slr1848	hisF	histidinol dehydrogenase	1.1.1.23	HISOL + 2 NAD -> HIS + 2 NADH

509	slr0091	hisG	aldehyde dehydrogenase	1.2.1.3	IMIDEHYDE + NAD -> IMIACE + NADH

510	slr0357	hisS	histidyl-tRNA synthetase	6.1.1.21	ATP + HIS + TRNA -> AMP + PPI + HHTRNA

Tyrosine metabolism

511	sll0402	aspH	aspartate aminotransferase	2.6.1.1	4HPP + GLU <-> TYR + AKG

512	sll0480	amo	putative aminotransferase	2.6.1.1	4HPP + GLU <-> TYR + AKG

513	slr0036	aspI	aspartate aminotransferase	2.6.1.1	4HPP + GLU <-> TYR + AKG

514	sll1713	hisC	histidinol-phosphate aminotransferase

2.6.1.9	4HPP + GLU <-> TYR + AKG

515	sll1958	hisC	histidinol phosphate aminotransferase

2.6.1.9	4HPP + GLU <-> TYR + AKG

516	slr0090	ppd	4-hydroxyphenylpyruvate dioxygenase

1.13.11.27	4HPP + O2 -> HOMOGEN + CO2

517	sll0990	fom	formaldehyde dehydrogenase (glutathione) / alcohol dehydrogenase

1.2.1.1	1.1.1.1	DIHPHETGLY + NAD <-> DIHMDDHY + NADH

Phenylalanine metabolism

518	sll0402	aspk	aspartate aminotransferase	2.6.1.1	PHE + AKG <-> PHPYR + GLU

519	sll0480	aspj	putative aminotransferase	2.6.1.1	PHE + AKG <-> PHPYR + GLU

520	slr0036	aspn	aspartate aminotransferase	2.6.1.1	PHE + AKG <-> PHPYR + GLU

521	sll1713	hisH	histidinol-phosphate aminotransferase

2.6.1.9	PHE + AKG <-> PHPYR + GLU

522	sll1958	hisC	histidinol phosphate aminotransferase

2.6.1.9	PHE + AKG <-> PHPYR + GLU

523	slr0090	ppd	4-hydroxyphenylpyruvate dioxygenase

1.13.11.27	PHPYR + O2 -> HYPHENACE + CO2

524	sll0828	nylA	6-aminohexanoate-cyclic-dimer hydrolase

3.5.1.4	PAD -> PAC + NH3

525	slr0091	ald10	aldehyde dehydrogenase	1.2.1.3	IACALD + NAD -> IAC + NADH

526	slr1884	trpS	tryptophanyl-tRNA synthetase	6.1.1.2	ATP + TRP + TRNA -> AMP + PPI + TRPTRNA

527	sll0828	nylA	6-aminohexanoate-cyclic-dimer hydrolase

3.5.1.4	IAD -> IAC + NH3

528	sll1987	cpx	catalase peroxidase catalase hpi	1.11.1.6	2 HAN + 2 O2 -> CINVALIN + 2 H2O2

529	sll1987	katG	catalase peroxidase catalase hpi	1.11.1.6	2 H2O2 -> O2

Phenylalanine tyrosine and tryptophan biosynthesis

530	sll0934	ccmA	phospho-2-dehydro-3-deoxyheptonate aldolase

2.5.1.54	E4P + PEP -> 3DDAH7P + PI

531	slr2130	aroB	3-dehydroquinate synthase	4.2.3.4	3DDAH7P -> DQT + PI

532	sll1112	aroQ	carbonic 3-dehydroquinase	4.2.1.10	DQT -> DHSK

533	slr0752	eno	enolase	4.2.1.11	DQT -> DHSK

534	slr1559	aroE	shikimate 5-dehydrogenase	1.1.1.25	DHSK + NADPH -> SME + NADP

535	sll1669	aroK	shikimate kinase	2.7.1.71	SME + ATP -> ADP + SME5P

536	slr0444	aroA	3-phosphoshikimate 1-carboxyvinyltransferase

2.5.1.19	SME5P + PEP -> 3PSME + PI

537	sll1747	aroC	chorismate synthase	4.2.3.5	3PSME -> PI + CHOR

538	slr0738	trpE	anthranilate synthase alpha-subunit	4.1.3.27	CHOR + GLN -> GLU + PYR + AN

539	slr1979	trpE	anthranilate synthase component I	4.1.3.27	CHOR + GLN -> GLU + PYR + AN

540	slr0055	trpG	anthranilate synthase component II	4.1.3.27	CHOR + NH3 -> AN + PYR

541	slr1867	trpD	anthranilate phosphoribosyltransferase

2.4.2.18	AN + PRPP -> PPI + NPRAN

542	sll0356	trpF	N-(5'-phosphoribosyl)anthranilate isomerase

5.3.1.24	NPRAN -> CPAD5P

543	slr0546	trpC	indole-3-glycerol phosphate synthase

4.1.1.48	CPAD5P -> CO2 + IGP

544	slr0966	trpA	tryptophan synthase alpha chain	4.2.1.20	IGP + SER -> T3P1 + TRP

545	slr0543	trpB	tryptophan synthase beta chain	4.2.1.20	IGP + SER -> T3P1 + TRP

546	sll0109	aroH	chorismate mutase	5.4.99.5	CHOR -> PHEN

547	slr2081	tyrA	prephenate dehydrogenase	1.3.1.12	PHEN + NADP -> 4HPP + CO2 + NADPH

548	slr2081	tyrA	prephenate dehydrogenase	1.3.1.12	PHEN + NAD -> 4HPP + CO2 + NADH

549	sll1662	pheA	prephenate dehydratase	4.2.1.51	PHEN -> CO2 + PHPYR

550	sll0454	pheS	phenylalanyl-tRNA synthetase	6.1.1.20	ATP + PHE + TRNA -> AMP + PPI + PHETRNA

551	sll1553	pheT	phenylalanyl-tRNA synthetase	6.1.1.20	ATP + PHE + TRNA -> AMP + PPI + PHETRNA

552	slr1031	tyrS	tyrosyl-tRNA synthetase	6.1.1.1	ATP + TYR + TRNA -> AMP + PPI + TYRTRNA

553	ssr1720	tyrS	tyrosyl tRNA synthetase	6.1.1.1	ATP + TYR + TRNA -> AMP + PPI + TYRTRNA

Urea cycle and metabolism of amino groups

554	slr2035	proB	glutamate 5-kinase	2.7.2.11	GLU + ATP -> ADP + GLUP

555	sll0373	proA	glutamate-5-semialdehyde dehydrogenase

1.2.1.41	GLUP + NADPH -> NADP + PI + GLUGSAL

556	sll0461	proA	gamma-glutamyl phosphate reductase

1.2.1.41	GLUP + NADPH -> NADP + PI + GLUGSAL

557	sll1883	argJ	glutamate N-acetyltransferase / amino-acid N-acetyltransferase

2.3.1.35	2.3.1.1	NAORN + GLU -> ORN + NAGLU

558	sll1883	argJ	glutamate N-acetyltransferase / amino-acid N-acetyltransferase

2.3.1.35	2.3.1.1	GLU + ACCOA -> COA + NAGLU

559	slr1898	argB	N-acetylglutamate kinase	2.7.2.8	NAGLU + ATP -> ADP + NAGLUP

560	sll0080	argC	N-acetyl-gamma-glutamyl-phosphate reductase

1.2.1.38	NAGLUP + NADPH -> NADP + PI + NAGLUS

561	slr1022	argD	acetylornithine aminotransferase	2.6.1.11	NAGLUS + GLU -> AKG + NAORN

Cyanoamino acid metabolism

562	sll1249	panC, kcy	pantoate--beta-alanine ligase / cytidylate kinase

6.3.2.1	2.7.4.14	PANT + bALA + ATP -> AMP + PPI + PNTO

563	slr0091	ald11	aldehyde dehydrogenase	1.2.1.3	BEAMPRAL + NAD -> bALA + NADH

564	sll2010	murD	UDP-N-acetylmuramoylalanine-D-glutamate ligase

6.3.2.9	UDPMURALA + DEXGLU + ATP -> ADP + PI + UALGLU

565	slr2079	glt2	glutaminase	3.5.1.2	DEXGLN -> DEXGLU + NH3

D-Alanine metabolism

566	slr1423	murC	UDP-N-acetylmuramate-alanine ligase

6.3.2.8	UDPMURAM + ALA + ATP -> ADP + PI + UDPMURALA

567	slr1874	ddlA	D-alanine--D-alanine ligase	6.3.2.4	2 DEXALA + ATP -> ADP + PI + ALAL

Glutathione metabolism

568	slr1269	ggt	gamma-glutamyltranspeptidase precursor

2.3.2.2	RGT -> GLU + ALAGLY

569	slr1269	ggt	gamma-glutamyltranspeptidase precursor

2.3.2.2	RGT -> CGLY + GLU

570	slr1843	zwf	glucose-6-phosphate 1-dehydrogenase

1.1.1.49	bDG6P + NADP <-> D6PGL + NADPH

571	slr1171	gpx1	glutathione peroxidase-like NADPH peroxidase glutathione peroxidase

1.11.1.9	2 RGT + H2O2 -> OGT

572	slr1992	gpx2	glutathione peroxidase-like NADPH peroxidase glutathione peroxidase

1.11.1.9	2 RGT + H2O2 -> OGT

573	sll0045	spsA	sucrose phosphate synthase	2.4.1.14	UDPG + F6P -> UDP + SUC6P

Starch and sucrose metabolism

574	sll1138	bet	beta-phosphoglucomutase homolog	5.4.2.6	bDG1P <-> bDG6P

575	slr1448	cscK	fructokinase	2.7.1.4	ATP + FRUBETA -> ADP + F6P

576	slr1176	glgC	glucose-1-phosphate adenylyltransferase

2.7.7.27	G1P + ATP -> PPI + ADPGLUC

577	sll0945	glgA	glycogen synthase	2.4.1.21	ADPGLC -> ADP + GLYCOGEN

578	sll1393	glgA	glycogen (starch) synthase	2.4.1.21	ADPGLC -> ADP + GLYCOGEN

579	sll0158	glgB	1 4-alpha-glucan branching enzyme	2.4.1.18	GLYCOGEN + PI -> G1P

580	sll1356	glgP	glycogen phosphorylase	2.4.1.1	GLYCOGEN + PI -> G1P

581	slr1367	glgY	glycogen phosphorylase	2.4.1.1	GLYCOGEN + PI -> G1P

582	sll1676	malQ	4-alpha-glucanotransferase	2.4.1.25	MLT -> 2 GLC

583	slr0983	rfbF	alpha-D-glucose-1-phosphate cytidylyltransferase

2.7.7.33	CTP + G1P -> PPI + CDPGLUC

584	slr0984	rfbG	CDP-glucose 4 6-dehydratase	4.2.1.45	CDPGLUC -> CDPDDGLU

N-Glycans biosynthesis

585	sll1540	"dpm1, sed3"	dolichyl-phosphate-mannose synthase

2.4.1.83	GDPMAN + DOLP -> GDP + DOLMANP

586	slr1943	"dpm1, sed3"	dolichyl-phosphate-mannose synthase

2.4.1.83	GDPMAN + DOLP -> GDP + DOLMANP

Aminosugars metabolism

587	sll0899	glmU	UDP-N-acetylglucosamine pyrophosphorylase / diamine N-acetyltransferase

2.7.7.23	2.3.1.57	UTP + NAGA1P <-> UDPNAGA + PPI

588	slr0624	glmW	UDP-N-acetylglucosamine 2-epimerase

5.1.3.14	UDPNAGA <-> NAMAN + UDP

589	sll0726	pgm	phosphoglucomutase	5.4.2.2	5.4.2.10	GA6P -> GA1P

590	sll1758	pgm2r	phosphoglucomutase	5.4.2.2	GA6P -> GA1P

591	slr0017	murA	UDP-N-acetylglucosamine 1-carboxyvinyltransferase

2.5.1.7	UDPNAGA + PEP <-> UDPNACEX + PI

592	slr1424	murB	UDP-N-acetylmuramate dehydrogenase

1.1.1.158	UDPNACEX + NADH -> NAD + UDPMURAM

593	slr1424	murC	UDP-N-acetylmuramate dehydrogenase

1.1.1.158	UDPNACEX + NADPH -> NADP + UDPMURAM

Lipopolysaccharide biosynthesis

594	sll0379	lpxA	acyl-[acyl-carrier-protein]-UDP-N-acetylglucosam ine o-acyltransferase

2.3.1.129	C140ACP + UDPNAG -> ACP + UDPG2AA

595	slr0015	lpxB	lipid A disaccharide synthase	2.4.1.182	LIPX + UDPG23A -> UDP + DISAC1P

596	sll1848	acy	1-acyl-sn-glycerol-3-phosphate acyltransferase

2.3.1.51	GL3P + 0.035 C140ACP + 0.102 C141ACP + 0.717 C160ACP + 0.142 C161ACP + 1.004 C181ACP -> 2 ACP + PA

Glycerolipid metabolism

597	slr0054	dgkA	diacylglycerol kinase	2.7.1.107	DAG + ATP -> ADP + PA

598	slr1369	cdsA	phosphatidate cytidylyltransferase	2.7.7.41	PA + CTP <-> CDPDG + PPI

599	sll1522	pgsA	CDP-diacylglycerol-glycerol-3-phosphate -3 phosphatidyltransferase

2.7.8.5	CDPDG + GL3P <-> CMP + PGP

600	slr0495	lipB	lipopeptide antibiotics iturin a biosynthesis protein

2.7.8.-	CDPDG + PG -> CMP + CL

601	slr1672	glpK	glycerol kinase	2.7.1.30	GL + ATP -> GL3P + ADP

602	sll1085	glpD	glycerol-3-phosphate dehydrogenase

1.1.99.5	GL3P + FAD -> T3P2 + FADH2

603	slr1755	gpsA	glycerol-3-phosphate dehydrogenase (NAD(P)+)

1.1.1.94	GL3P + NADP <-> T3P2 + NADPH

604	slr1167	gldA	glycerol dehydrogenase	1.1.1.6	GL + NAD <-> GLYN + NADH

605	sll0990	form2	formaldehyde dehydrogenase (glutathione) / alcohol dehydrogenase

1.2.1.1	1.1.1.1	GL + NAD <-> GLYALDHY + NADH

606	slr0942	form3	aldehyde reductase	1.1.1.2	GL + NADP <-> GLYALDHY + NADPH

Sphingoglycolipid metabolism

607	slr0091	form4	aldehyde dehydrogenase	1.2.1.3	GLYALDHY + NAD <-> GLYCERATE + NADH

608	slr0091	form5	aldehyde dehydrogenase	1.2.1.3	LACAL + NADH <-> PROP12D + NAD

609	sll1969	lipA	lipase homolog	3.1.1.3	TAGLY -> DAGLY + a Carboxylate

610	sll1969	lipA	lipase homolog	3.1.1.3	TAGLY -> DAGLY + a fatty acid anion

611	sll1383	"suhB, ssyA"	extragenic suppressor SuhB

3.1.3.25	MI1P -> MYOI + PI

612	sll0635	thiE	thiamin-phosphate pyrophosphorylase

2.5.1.3	THZP + AHMPP -> THMP + PPI

Thiamine metabolism

613	slr1787	thiB	putative thiamine-monophosphate kinase

2.7.4.16	THMP + ATP <-> TPP + ADP

614	slr0118	thiC	thiamin biosynthesis protein ThiC	AIR -> AHM

615	slr0633	thiG	ThiG protein	DTP + TYR + CYS -> THZ + HBA + CO2

616	ssr0102	ycf40	thiamin biosynthesis ThiS	DTP + TYR + CYS -> THZ + HBA + CO2

Riboflavin metabolism

617	sll1894	ribA	GTP cyclohydrolase II / 3 4-dihydroxy-2-butanone 4-phosphate synthase (dhbp synthase)

3.5.4.25	GTP -> D6RP5P + FOR + PPI

618	slr0066	ribD	diaminohydroxyphosphoribosylaminopyrimidine deaminase / 5-amino-6-(5-phosphoribosylamino)uracil reductase	3.5.4.26	1.1.1.193	D6RP5P -> A6RP5P + NH3

619	slr0066	ribD	diaminohydroxyphosphoribosylaminopyrimidine deaminase / 5-amino-6-(5-phosphoribosylamino)uracil reductase	3.5.4.26	1.1.1.193	A6RP5P + NADPH -> A6RP5P2 + NADP

620	sll0300	ribC	riboflavin synthase alpha chain	2.5.1.9	2 D8RL -> RIBOFLAV + A6RP

621	sll1282	ribH	riboflavin synthase beta chain	2.5.1.9	A6RP5P2 + R5P -> D8RL + PI

622	slr1882	ribF	riboflavin kinase / FMN adenylyltransferase

2.7.1.26	2.7.7.2	RIBOFLAV + ATP -> FMN + ADP

623	slr1882	ribF	riboflavin kinase / FMN adenylyltransferase

2.7.1.26	2.7.7.2	FMN + ATP -> FAD + PPI

624	sll1108	surE	survival protein SurE homolog	3.1.3.2	FMN -> RIBOFLAV + PI

Vitamin B6 metabolism

625	sll1440	pdxH	pyridoxamine 5-phosphate oxidase	1.4.3.5	PDLA5P + O2 -> PL5P + H202 + NH3

626	sll1440	pdxH	pyridoxamine 5-phosphate oxidase	1.4.3.5	P5P + O2 <-> PL5P + H2O2

627	sll1440	pdxH	pyridoxamine 5-phosphate oxidase	1.4.3.5	PYRDX + O2 <-> PL + H2O2

628	sll1440	pdxH	pyridoxamine 5-phosphate oxidase	1.4.3.5	PL + O2 + NH3 <-> PDLA + H2O2

629	sll0660	pdxA	pyridoxal phosphate biosynthetic protein PdxA

1.1.1.262	PHT + 1DX5P -> P5P + CO2

630	slr1779	pdxJ	pyridoxal phosphate biosynthetic protein PdxJ	PHT + 1DX5P -> P5P + CO2

631	sll1172	thrC	threonine synthase	4.2.3.1	PHT -> 4HLT + PI

632	sll1688	thrC	threonine synthase	4.2.3.1	PHT -> 4HLT + PI

Nicotinate and nicotinamide metabolism

633	sll0631	nadB	L-aspartate oxidase	1.4.3.16	ASP + O2 -> IMINASP + H2O2

634	sll0622	nadA	quinolinate synthetase	IMINASP + T3P2 -> PI + QA

635	slr0936	nadC	nicotinate-nucleotide pyrophosphorylase (carboxylating)

2.4.2.19	QA + PRPP -> NAMN + CO2 + PPI

636	slr0787	nadF	nicotinamide-nucleotide adenylyltransferase / ADP compounds hydrolase

2.7.7.1	3.6.1.-	NAMN + ATP <-> NAAD + PPI

637	slr1916	nadG	nicotinate-nucleotide adenylyltransferase

2.7.7.18	NICAMRIB + ATP <-> PPI + NAAD

638	slr1691	nadE	NH(3)-dependent NAD(+) synthetase

6.3.5.1	NAAD + ATP + NH3 -> NAD + AMP + PPI

639	slr1239	pntA	NAD(P) transhydrogenase subunit alpha

1.6.1.2	NADPH + NAD <-> NADP + NADH

640	slr1434	pntB	NAD(P) transhydrogenase subunit beta

1.6.1.2	NADPH + NAD <-> NADP + NADH

Pantothenate and CoA biosynthesis

641	sll1415	pntC	probable inorganic polyphosphate/ATP-NAD kinase 2

2.7.1.23	ATP + NAD -> ADP + NADP

642	slr0400	pntD	probable inorganic polyphosphate/ATP-NAD kinase 1

2.7.1.23	ATP + NAD -> ADP + NADP

643	slr0526	panB	3-methyl-2-oxobutanoate hydroxymethyltransferase

2.1.2.11	OMVAL + METTHF -> THF + AKP

644	slr0847	kdtB	pantetheine-phosphate adenylyltransferase

2.7.7.3	4PPNTE + ATP <-> DPCOA + PPI

645	slr0553	dep	dephospho-CoA kinase	2.7.1.24	ATP + DPCOA -> COA + ADP

Biotin metabolism

646	slr0917	bioF	7-keto-8-aminopelargonic acid synthetase

2.3.1.47	ALA + CHCOA <-> CO2 + COA + AONA

647	slr0523	bioD	dethiobiotin synthetase	6.3.3.3	CO2 + DANNA + ATP <-> DTB + PI + ADP

648	slr1364	bioB	biotin synthetase	2.8.1.6	DTB + CYS <-> BT

Folate biosynthesis

649	slr0426	folE	GTP cyclohydrolase I	3.5.4.16	GTP -> FOR + AHTD

650	sll0672	pacL	cation-transporting p-type ATPase PacL

3.6.1.-	ATP <-> ADP + PI

651	sll1076	pacL	cation-transporting p-type ATPase PacL

3.6.1.-	ATP <-> ADP + PI

652	folB	dihydroneopterin aldolase	4.1.2.25	DHP -> AHHMP + GLAL

653	slr1626	folK	7 8-dihydro-6-hydroxymethylpterin-pyrophosphokinase

2.7.6.3	AHHMP + ATP -> AMP + AHHMD

654	slr1093	folP	dihydropteroate synthase	2.5.1.15	PABA + AHHMD -> PPI + DHPT

655	slr2026	folC	folylpolyglutamate synthase / dihydrofolate synthase

6.3.2.17	DHPT + ATP + GLU -> ADP + PI + DHF

656	sll1612	dedC	folylpolyglutamate synthase / dihydrofolate synthase

6.3.2.17	ATP + GLU <-> ADP + PI + THFG

657	sll1612	dedD	6-pyruvoyl tetrahydrobiopterin synthase

4.2.3.12	AHTD -> PPP + 6PYRHYTER

658	slr0078	dedE	sepiapterine reductase	1.1.1.153	DHBP + NADP <-> SPT + NADPH

659	sll0330	folD	methylenetetrahydrofolate dehydrogenase (NADP+) / methenyltetrahydrofolate cyclohydrolase

1.5.1.5	3.5.4.9	METTHF + NAD -> METHF + NADH

Porphyrin and chlorophyll metabolism

660	sll0753	purN	phosphoribosylglycinamide formyltransferase

2.1.2.2	GAR + METHF -> THF + FGAR

661	slr0477	gcvT	aminomethyltransferase	2.1.2.10	THF + SAP <-> METTHF + NH3 + DILIPS

662	sll0171	gcvT	aminomethyltransferase	2.1.2.10	METHF -> 5FORMS

663	sll0171	hemA	glutamyl-tRNA reductase	1.2.1.-	GTRNA + NADPH -> GSA + NADP

664	slr1808	hemL	glutamate-1-semialdehyde 2 1-aminomutase

5.4.3.8	GLLUGSAL1 -> ALAV

665	sll0017	hemB	porphobilinogen synthase (5-aminolevulinate dehydratase)

4.2.1.24	2 ALAV -> PBG

666	sll1994	hemC	porphobilinogen deaminase (hydroxymethylbilane synthase preuroporphyrinogen synthase)

2.5.1.61	4 PBG -> HMB + 4 NH3

667	slr1887	cobA	a fusion protein between uroporphyrinogen-III C-methyltransferase (CobA/CorA) and uroporphyrinogen-III synthase (HemD)	4.2.1.75	2.1.1.107	HMB -> UPRG

668	sll0166	cysG	uroporphyrin-III C-methyltransferase

2.1.1.107	2 SAM + UPRG + NAD -> 2 SAH + PC2 + NADH

669	sll0378	hemE	uroporphyrinogen decarboxylase	4.1.1.37	UPRG -> CPP + 4 CO2

670	slr0536	hemE	uroporphyrinogen decarboxylase	4.1.1.37	UPRGI -> CPPI + 4 CO2

671	slr0536	hemF	coproporphyrinogen III oxidase aerobic (oxygen dependant)

1.3.3.3	O2 + CPP -> 2 CO2 + PPHG

672	sll1185	hemN1	coproporphyrinogen III oxidase anaerobic (oxygen independant)

1.3.3.3	O2 + CPP -> 2 CO2 + PPHG

673	sll1876	hemN2	coproporphyrinogen III oxidase anaerobic (oxygen independant)

1.3.3.3	O2 + CPP -> 2 CO2 + PPHG

674	sll1917	hemH	ferrochelatase	4.99.1.1	PPIX -> PTH

675	slr0839	cbiL	S-adenosyl-methionine:precorrin-2 methyltransferase

2.1.1.130	COPRECOR2 + SAM -> COPRECOR3 + SAH

676	slr1879	cbiH	Precorrin-3B C17-methyltransferase (Precorrin-3 methyltransferase)

2.1.1.131	COPRECOR3 + SAM -> COPRECOR4 + SAH

677	slr0969	cbiF	precorrin-4 C11-methyltransferase	2.1.1.133	COPRECOR4 + SAM -> COPRECOR5 + SAH

678	slr0239	cbia	precorrin-6X reductase	1.3.1.54	COPRECOR6 + NADPH -> DICOCOR6 + NADP

679	slr0252	cbiE	precorrin-6y C5 15-methyltransferase (decarboxylating)

2.1.1.132 1.-.-.-	DICOCOR6 + 2 SAM -> COPRECOR8 + 2 SAH + CO2

680	sll0099	corR	cobalt-dependent transcriptional regulator

5.4.1.2	COPRECOR8 -> COBY

681	sll0794	cbiC	precorrin isomerase precorrin-8X methylmutase

5.4.1.2	COPRECOR8 -> COBY

682	sll0916	cbiD	precorrin-8X methylmutase	5.4.1.2	COPRECOR8 -> COBY

683	slr1467	cobM	cobalamin biosynthesis protein CobN

4.99.1.-	SHCL -> SHEME

684	sll1890	cobN	cobalt-chelatase subunit CobN catalysing insertion of cobalt ion into hydrogenobyrinic acid a c-diamide

4.99.1.-	SHCL -> SHEME

685	slr1211	cobO	cob(I)alamin adenosyltransferase	2.5.1.17	COBIALA + ATP -> COBCOENZ + PPI + PI

686	slr0260	bchH	Magnesium protoporphyrin IX chelatase subunit H

6.6.1.1	PPIX + ATP + MAGGIE -> ADP + PI + MAGGIEPOR

687	slr1055	chlD	Magnesium protoporphyrin IX chelatase subunit D

6.6.1.1	PPIX + ATP + MAGGIE -> ADP + PI + MAGGIEPOR

688	slr1777	chlI	Magnesium protoporphyrin IX chelatase subunit I

6.6.1.1	PPIX + ATP + MAGGIE -> ADP + PI + MAGGIEPOR

689	slr1030	chlM	Mg-protoporphyrin IX methyl transferase

2.1.1.11	MAGGIEPOR + SAM -> MAGPORMOE + SAH

690	slr0525	chlN	3-octaprenyl-4-hydroxybenzoate carboxy-lyase UbiD

4.1.1.-	O4HBZ -> CO2 + 2OPPP

Ubiquinone biosynthesis

691	sll0936	ubiX	3-octaprenyl-4-hydroxybenzoate carboxy-lyase

4.1.1.-	O4HBZ -> CO2 + 2OPPP

692	slr1099	menF	isochorismate synthase	5.4.4.2	CHOR <-> CHORISO

693	slr0817	menD	2-oxoglutarate decarboxylase / 2-succinyl-6-hydroxy-2 4-cyclohexadiene-1-carboxylate synthase

4.1.1.71	4.1.3.-	AKG + TPP -> SUCSALTDA + CO2

694	sll0603	menC	O-succinylbenzoate-CoA synthase	4.2.1.-	SHCHC -> OSB

695	sll0409	menE	O-succinylbenzoic acid-CoA ligase	6.2.1.26	OSB + ATP + COA -> OSBCOA + AMP + PPI

696	slr0492	menB	1 4-dihydroxy-2-naphthoate synthase

4.1.3.36	OSBCOA -> DHNA + COA

697	sll1127	ndhB	NADH dehydrogenase I chain N	1.6.5.3	UBIQ <-> UBIQOL

698	sll0223	gpp1	GPP + IPPP -> FPP + PPI

Added Reactions from CyanoBase (to complete pathways)

699	fpp1	FPP + IPPP -> GERAPP + PPI

700	slr1255	crtB, pys	phytoene synthase	2.5.1.32	2 GERAPP -> PREPHYPP + PPI

701	PAN5	"PUTATIVE KETOPANTOATE REDUCTASE (2-DEHYDROPANTOATE 2-REDUCTASE) INVOLVED IN COENZYME A SYNTHESIS, HAS SIMILARITY TO CBS2P, KETOPANTOATE REDUCTASE"

1.1.1.169	AKP + NADPH <-> PANT + NADP

702	COAA	Pantothenate kinase	2.7.1.33	PNTO + ATP -> 4PPNTO + ADP

703	PCLIG	Phosphopantothenate-cysteine ligase

6.3.2.5	4PPNTO + CYS + CTP -> 4PPNCYS + PI + CDP

704	PCDCL	Phosphopantothenate-cysteine decarboxylase

4.1.1.36	4PPNCYS -> 4PPNTE + CO2

705	mis5	2.3.1.157	GA1P + ACCOA <-> COA + NAGA1P

706	METF	Methylene tetrahydrofolate reductase

1.7.99.5	METTHF + NADH -> MTHF + NAD

707	mis7	2.7.1.89	THIAMIN + ATP -> THMP + ADP

708	THIB	Thiamin phosphate synthase	2.7.1.50	2.5.1.3	AHMPP + ATP + T3P1 + PYR -> THMP + PPI

709	THI20	"BIPARTITE PROTEIN CONSISTING OF N-TERMINAL HYDROXYMETHYLPYRIMIDINE PHOSPHATE (HMP-P) KINASE	DOMAIN, NEEDED FOR THIAMINE BIOSYNTHESIS, FUSED TO C-TERMINAL PET18P-LIKE DOMAIN OF INDETERMINANT	FUNCTION"	2.7.1.49	2.7.4.7	AIR + 2 ATP -> AHMPP + 2 ADP

710	mis10	6.2.1.9	MAL + ATP + COA -> ADP + PI + MCA

711	mis11	4.1.3.24	MCA <-> ACCOA + GLX

712	ACEA	Isocitrate lyase	4.1.3.1	ICIT -> GLX + SUCC

713	GSH1	GAMMA-GLUTAMYLCYSTEINE SYNTHETASE

6.3.2.2	CYS + GLU + ATP -> GC + PI + ADP

714	PHA2	PREPHENATE DEHYDRATASE	4.2.1.51	PHEN -> PHPYR + CO2

715	PUTA1	Proline dehydrogenase	1.5.99.8	PRO + FAD -> PHC + FADH2

716	YGJG	Ornithine transaminase	2.6.1.13	ORN + AKG -> GLUGSAL + GLU

717	HIS2	HISTIDINOLPHOSPHATASE	3.1.3.15	HISOLP -> HISOL +PI

718	HIS7	GLUTAMINE AMIDOTRANSFERASE:CYCLASE

2.4.2.-	PRLP + GLN -> GLU + AICAR

719	DAPD	Tetrahydrodipicolinate succinylase	2.3.1.117	PIP26DX + SUCCOA -> NS2A6O + COA

720	DAPE	Succinyl diaminopimelate desuccinylase

3.5.1.18	NS26DP -> D26PIM + SUCC

721	mis21	Thymidylate synthase	2.1.1.148	DUMP + METTHF -> DHF + DTMP

722	GAL1	galactokinase	2.7.1.6	GLAC + ATP -> GAL1P + ADP

723	GAL7	UDPglucose--hexose-1-phosphate uridylyltransferase

2.7.7.12	UDPG + GAL1P <-> G1P + UDPGAL

724	GALU2	UTP--Glucose 1-Phosphate Uridylyltransferase

2.7.7.9	G1P + UTP <-> UDPG + PPI

725	FTHFLGS	the cytoplasmic trifunctional enzyme C1-tetrahydrofolate synthase

6.3.4.3	THF + FOR + ATP -> ADP + PI + FTHF

726	DEOD1	Xanthosine phosphorylase, Purine nucleotide phosphorylase

2.4.2.1	AD + R1P <-> PI + ADENOS

727	AMD1	AMP deaminase	3.2.2.4	AMP -> AD + R5P

728	RBK1	Ribokinase	2.7.1.15	RIB + ATP -> R5P + ADP

729	RBK1	Ribokinase	2.7.1.15	DRIB + ATP -> DR5P + ADP

730	COR1	ubiquinol-cytochrome c oxidoreductase subunit 7 (14 kDa)

1.10.2.2	2 FERI + UBIQOL <-> 2 FERO + UBIQ

Biomass Equations:

731

a. Heterotrophic growth:	0.882 G6P + 0.406 E4P + 0.238 GAP + 1.53 PEP + 2.64 PYR + 4.64 ACA + 1.23 OAA + 1.04 AKG + 0.399 R5P + 2.82	NAD + 27.22 NADPH + 39.21 ATP -> BIOMASS_H + 1.8346 CO2 + 4.64 COA + 39.21 ADP + 2.82 NADH + 27.22 NADP

b. Photoautotrophic growth:

1.191 G6P + 0.501 E4P + 0.133 GAP + 1.205 3PG + 1.002 PEP + 1.197 PYR + 3.727 ACA + 2.039 OAA + 1.233 AKG + 0.16 SUCCOA +	0.715 R5P + 2.82 NAD + 49.06 NADPH + 53.35 ATP -> BIOMASS_P + 1.017 CO2 + 3.887 COA + 53.35 ADP + 2.82 NADH + 49.06	NADP + 0.683 FUM + 0.103 AC

c. Mixotrophic growth:

1.228 G6P + 0.376 E4P + 0.208 GAP + 1.42 PEP + 2.44 PYR + 3.96 ACA + 1.14 OAA + 0.886 AKG + 0.382 R5P + 2.82 NAD + 29.01	NADPH + 38.89 ATP -> BIOMASS_M + 1.834 CO2 + 3.96 COA + 38.89 ADP + 2.82 NADH + 29.01 NADP

Lighting Reaction:

732	2.0 NADP + 2.6 ADP + 2.6 PI + 0.125 APF + HEXT -> 2.0 NADPH + 2.60 ATP +1.0 O2

Insertion of the folowing two genes for ethanol-producing mutant

733	pdc	pyruvate decarboxylase	4.1.1.1	PYR -> ACAL + CO2

734	adh	alcohol dehydrogenase isoenzyme IV

1.1.1.1	ACAL + NADH <-> ETH + NAD

Transport Equations:

735	GLUxt + ATP -> GLU + ADP + PI

736	FRUxt -> FRU

737	MANxt -> MAN1P

738	GLACxt -> GLAC

739	SUCxt <-> HEXT + SUC

740	MALxt <-> MAL

741	AKGxt <-> AKG

742	APFxt -> APF

743	GLCxt -> GLC

744	GLxt <-> GL

745	LACxt <-> HEXT + LAC

746	MLTxt + PEP -> MLT6P + PYR

747	MLTxt + HEXT <-> MLT

748	HCO3xt <-> CO2

749	ALAxt + ATP -> ALA + ADP + PI

750	ARGxt + ATP -> ARG + ADP + PI

751	ARGxt + HEXT <-> ARG

752	ASNxt + NADH <-> NAD + ASN

753	ASNxt + NADH -> NAD + ASN

754	ASPxt + NADH <-> NAD + ASP

755	ASPxt + NADH +1.0000000 NAD -> ASP

756	CYSxt + ATP -> CYS + ADP + PI

757	DALAxt + ATP -> DALA + ADP + PI

758	GLYCTxt + HEXT <-> GLYCOLATE

759	GLYCOxt + HEXT <-> GLYCOGEN

760	GLYxt + NADPH <-> NADP + GLY

761	MDAPxt + ATP -> MDAP + ADP + PI

762	GABAxt + ATP -> GABA + ADP + PI

763	GLNxt + NADH -> NAD + GLN

764	GLYxt + NADH -> NAD + GLY

765	HISxt + ATP -> HIS + ADP + PI

766	ILExt + ATP -> ILE + ADP + PI

767	LEUxt + ATP -> LEU + ADP + PI

768	LYSxt + HEXT <-> LYS

769	LYSxt + ATP + LYS -> ADP + PI

770	METxt + ATP -> MET + ADP + PI

771	ORNxt + ATP -> ORN + ADP + PI

772	ORNxt + HEXT <-> ORN

773	PHExt + HEXT <-> PHEN

774	PROxt + HEXT <-> PRO

775	PROxt + ATP -> PRO + ADP + PI

776	SERxt + NADH <-> NAD + SER

777	SERxt + NADH -> NAD + SER

778	THRxt + ATP -> THR + ADP + PI

779	THRxt + HEXT -> THR

780	TRPxt + HEXT <-> TRP

781	TYRxt + HEXT <-> TYR

782	URAxt + HEXT -> URA

783	CYTSxt + HEXT -> CYTS

784	NITRAxt + HEXT -> NITRA

785	ADxt + HEXT -> AD

786	GNxt + HEXT <-> GN

787	DTxt + HEXT -> DT

788	DGxt + HEXT -> DG

789	DCxt + HEXT -> DC

790	DUxt + HEXT -> DU

791	DTxt + HEXT -> DT

792	DCxt + HEXT -> DC

793	DTxt + HEXT -> DT

794	DGxt + HEXT -> DG

795	DCxt + HEXT -> DC

796	ACxt + HEXT <-> AC

797	FORxt <-> FOR

798	ETHxt <-> ETH

799	SUCCxt + HEXT <-> SUCC

800	PYRxt + HEXT <-> PYR

801	UREAxt + HEXT <-> UREA

802	NH3xt + HEXT <-> NH3

803	NH3xt + ATP <-> NH3 + ADP + PI

804

804	Kxt + ATP -> K + ADP + PI

805	Kxt + HEXT <-> K

806	SLFxt + ATP <-> SLF + ADP + PI

807	PIxt + ATP -> ADP + 2 PI

808	PIxt + HEXT <-> PI

809	GL3Pxt + PI -> GL3P

810	CITxt + HEXT -> CIT

811	FUMxt + HEXT <-> FUM

812	AKGxt + HEXT <-> AKG

813	PNTOxt + HEXT <-> PNTO

814	O2xt <-> O2

815	CO2xt <-> CO2

816	ATP -> ADP + PI

817	GLALxt <-> GLAL

818	ACALxt <-> ACAL

819	ATTxt -> ATT

820	VALxt + ATP <-> VAL + ADP + PI

821	PAPxt <-> PAP

822	THYxt <-> THY + HEXT

823	GA6Pxt <-> GA6P

824	SUCSxt <-> SUCS

825	GALACxt <-> GALAC

826	AONAxt <-> HEXT + AONA

827	OGTxt ->0 OGT

828	THMxt -> HEXT + THIAMIN

829	RFLAVxt <-> HEXT + RIBOFLAV

830	PYRDXxt <-> HEXT + PYRDX

831	NAD -> NADH

**"* Note: the suffix "xt" for the metabolites means "transport process" or "exchange flux"**
